# Tumor-penetrating peptide for systemic targeting of Tenascin-C

**DOI:** 10.1038/s41598-020-62760-y

**Published:** 2020-04-02

**Authors:** Prakash Lingasamy, Allan Tobi, Kaarel Kurm, Sergei Kopanchuk, Aleksander Sudakov, Markko Salumäe, Tõnu Rätsep, Toomas Asser, Rolf Bjerkvig, Tambet Teesalu

**Affiliations:** 1 Laboratory of Cancer Biology, Institute of Biomedicine and Translational Medicine, University of Tartu, Tartu, Estonia; 2 Cancer Research Center, Sanford Burnham Prebys Medical Discovery Institute, La Jolla, California USA; 3 Center for Nanomedicine and Department of Cell, Molecular and Developmental Biology, University of California, Santa Barbara, Santa Barbara, California USA; 4 Department of Neurosurgery, Tartu University Hospital, Tartu, Estonia; 5 Department of Biomedicine Translational Cancer Research, University of Bergen, Bergen, Norway; 6 Oxford Nanopore Technologies Ltd., Oxford, UK; 7 Institute of Chemistry, University of Tartu, Tartu, Estonia

**Keywords:** Targeted therapies, Cancer imaging, Cancer microenvironment, Cancer therapy, CNS cancer

## Abstract

Extracellular matrix in solid tumors has emerged as a specific, stable, and abundant target for affinity-guided delivery of anticancer drugs. Here we describe the homing peptide that interacts with the C-isoform of Tenascin-C (TNC-C) upregulated in malignant tissues. TNC-C binding PL3 peptide (amino acid sequence: AGRGRLVR) was identified by *in vitro* biopanning on recombinant TNC-C. Besides TNC-C, PL3 interacts via its C-end Rule (CendR) motif with cell-and tissue penetration receptor neuropilin-1 (NRP-1). Functionalization of iron oxide nanoworms (NWs) and metallic silver nanoparticles (AgNPs) with PL3 peptide increased tropism of systemic nanoparticles towards glioblastoma (GBM) and prostate carcinoma xenograft lesions in nude mice (eight and five-fold respectively). Treatment of glioma-bearing mice with proapoptotic PL3-guided NWs improved the survival of the mice, whereas treatment with untargeted particles had no effect. PL3-coated nanoparticles were found to accumulate in TNC-C and NRP-1-positive areas in clinical tumor samples, suggesting a translational relevance. The systemic tumor-targeting properties and binding of PL3-NPs to the clinical tumor sections, suggest that the PL3 peptide may have applications as a targeting moiety for the selective delivery of imaging and therapeutic agents to solid tumors.

## Introduction

The microenvironment in solid tumors is shaped by concerted actions of tumor cells and cells in tumor stroma (endothelial cells, immune cells, and fibroblasts). All these cells deposit and remodel tumor extracellular matrix (ECM) – a complex meshwork of structural proteins and bioactive compounds. In addition to the structural role, the tumor ECM modulates invasion and distant spreading of tumor cells, tumor angiogenic status, and immune microenvironment, thereby plays a critical role(s) in tumor initiation, progression, and metastasis^1^. The molecular composition of tumor ECM reflects its type, origin, and physiological status^2^. Tumor ECM is rich in components expressed in developing and/or regenerating tissues that are typically absent or expressed at a lower level in quiescent healthy adult tissues and organs^3^. Tenascin C (TNC) is an ECM glycoprotein that is upregulated during normal tissue repair and in many human malignancies and plays important roles in tumor neovascularization, modulation of adhesion, and local and distant motility of malignant cells, and modulation of tumor immune status^4^. Several affinity ligands of TNC have been developed for targeting payloads to solid tumors, such as G11 single-chain antibody^5^, aptamers^6^, FH peptide^7^, and PL1 peptide^8^. TNC targeting antibodies are in phase I/II/III clinical trials as affinity targeting modules for cytotoxic or immunomodulatory payloads for therapy of solid tumors including metastatic renal cell carcinoma, metastatic melanoma, pancreatic carcinoma, acute myeloid leukemia, sarcoma and breast carcinoma, lung cancer, colorectal cancer, and glioma^4^. Importantly, malignant transformation is not only able to increase the expression of tenascin-C, but also influence its qualitative composition by modulating the splicing to result in different proportion of its isoforms. In particular, 91-amino acid alternatively spliced type-III homology domain of Tenascin-C FnIII C (TNC-C) has a tumor-restricted expression pattern^5,8,9^. The TNC-C is abundant in the glial tumors of the CNS, as well as in various carcinomas^10–12^. Overexpression of TNC-C correlates with tumor grade and is associated with poor prognosis^12,13^.

Peptides have several advantages over antibodies as tumor-targeting vehicles, including small size (and thus straightforward synthesis, eliminating the need for antibody-protein engineering), low immunogenicity, moderate cost, biocompatibility, and low-micromolar affinity range that circumvents the affinity site barrier^14–16^. Small peptides as targeting elements are particularly relevant in the context of nanomaterials, where even small molecule targeting ligands with weak affinity can through multivalent interactions significantly enhance target-specific avidity (by up to 4 orders of magnitude), and thus the affinity of the final material is readily tunable^17^.

Here we set out to develop peptidic affinity ligands for targeting TNC-C in solid tumors. Using peptide phage display, we identified an octameric homing peptide, PL3, that interacts with TNC-C *in vitro* and *in vivo*. In addition, PL3 peptide was able to interact with cell- and tissue penetration receptor NRP-1. Systemic PL3-guided nanoparticles accumulated in tumor xenografts implanted in mice. These nanoparticles were useful for tumor detection, imaging and served as a tumor-seeking carrier for a proapoptotic peptide anticancer payload. Our study suggests applications for PL3-targeted compounds and nanoparticles for improved detection and therapy of solid tumors.

## Materials and Methods

### Materials

Phosphate-buffered saline (PBS) was purchased from Lonza (Verviers, Belgium). K_3_[Fe(CN)_6_], HCl, isopropanol, Triton-X, Tween-20, CHCl3, MeOH, Isopropyl β-D-1-thiogalactopyranoside (IPTG) and dimethylformamide (DMF) were purchased from Sigma-Aldrich (Munich, Germany). Cys-fluorescein (FAM)-PL3 and Cys-FAM peptides with 6-aminohexanoic acid spacer were purchased from TAG Copenhagen (Denmark). PL3-_D_(KLAKLAK)_2_ and _D_(KLAKLAK)_2_ peptides were synthesized using Fmoc/t-Bu chemistry on a microwave-assisted automated peptide synthesizer (Liberty; CEM Corporation, Matthews, NC, USA). High-performance liquid chromatography (HPLC) was used to purify the peptides with 0.1% trifluoroacetic acid (TFA) in acetonitrile-water mixtures to 90%–95% purity. The peptides were validated by quadrupole time-of-flight (Q-TOF) mass spectral analysis^18^. CF647 amine dye was purchased from Biotium (Hayward, CA, USA).

### Cell lines and experimental animals

The U87-MG (human glioblastoma, HTB-14) cells, PC3 (prostate carcinoma, CRL1435) cells, PPC1 (primary prostate carcinoma-1) cells were purchased from ATCC. Murine WT-GBM glioblastoma cells were a kind gift from Gabriele Bergers (UCSF, USA) and P3, P8, P13 stem cell-like, P22, NCH421K cells were a kind gift from Rolf Bjerkvig, (University of Bergen, Norway), and M21 melanoma cells were the gift of David Cheresh (USA). Cells and tumors were prepared as described previously^19–21^.

Athymic nude mice (Hsd/Athymic Fox1 nu Harlan) were purchased from Envigo (Netherlands) and maintained under standard housing conditions of the Animal Facility of the Institute of Biomedicine and Translational Medicine, University of Tartu (Tartu, Estonia). For orthotropic GBM tumor models, we used NCH421K, P13, and P3 stem cell-like, WT-GBM cells. The individual GBM cells around 2–3 × 10^5^ in 3 μL PBS were intracranially implanted into mice brain 2 mm right and 1 mm anterior to the bregma. U87-MG and PC3 subcutaneous models were induced by injecting 2–9 × 10^6^ cells in 100 µl PBS subcutaneously into the right flank of 11–15 week old male and female nude mice. All animal experiments were performed in accordance with the procedures approved by the Committee of Animal Experimentation of Estonian Ministry of Agriculture, permits #42 and #48.

### Peptide phage biopanning

For biopanning on recombinant human TNC-C, we used NNK-encoded CX7C and X7 peptide T7 phage libraries with diversity ~5 × 10^8^. Throughout our screens, the selected phages were amplified following plate amplification protocol^22^. The 1^st^ and 4^th^ rounds of biopanning were performed on TNC-C immobilized on Costar 96-Well ELISA plate (#3590, Corning Life Sciences, Tewksbury, MA, USA). Briefly, the multiwell plates were coated with 20 µg/ml recombinant TNC-C in 100 µl of PBS overnight at 4 °C, followed by blocking with 1% bovine serum albumin (BSA) in PBS overnight at 4 °C. The phage library (5 × 10^8^ pfu in 100 µl of PBS-BSA) was incubated overnight at 4 °C, followed by 6 washes with PBS containing 1% BSA and 0.1% Tween-20 (washing buffer) to remove background phages, and by phage rescue and amplification in E. *coli* strain BLT5403 (Novagen, EMD Biosciences, MA, USA)^8^. The subsequent rounds of selection were performed on Ni-NTA Magnetic Agarose Beads (QIAGEN, Hilden, Germany) coated with His-6X tagged TNC-C (30 µg/10 µl beads) at room temperature for 1 h in 400 µl of PBS. The TNC-C beads were washed 3 times with washing buffer, followed by incubation with phages in (5 × 10^8^ pfu in 100 µl in washing buffer) at room temperature for 1 h. The background phages were removed by rinsing 6 times with washing buffer, and the bound phages were eluted with 1 ml of PBS containing 500 mM Imidazole and 0.1% NP40.

The eluted phages were titered and amplified for a next round of selection. After 5 rounds of selection, peptide-encoding DNA from a set of 48 phage clones was subjected to Sanger sequencing of peptide-encoding phage DNA^18,22^. For cell-free binding studies with individual phage clones were incubated with Ni-NTA magnetic beads coated with hexahistidine-tagged TNC-C as above. RPARPAR phage binding to NRP-1-coated beads was used as a positive control^23^. Phage clones displaying heptaglycine peptide (GGGGGGG, G7), or insertless phage clones were used as negative controls.

### Fluorescence polarization assay

Fluorescence anisotropy (FA) saturation binding experiments were set up as described previously^24,25^. The experiments were carried out in Dulbecco’s Phosphate Buffer Saline (Sigma-Aldrrich, Cat# D8662) with the addition of 0.1% Pluronic F-127 (Sigma-Aldrrich, Cat#P2443) in a final volume of 100 μl using 96‐well half area, flat‐bottom polystyrene NBS multiwell plates (Corning, Cat# 3686). The different concentrations of proteins (0–112 µM NRP1 or 0–275 µM TNC-C) were added to a fixed concentration (0.66 µM) of FAM-Cys-PL3 fluorescent ligand (KJ Ross-Petersen aps). The total and non‐specific binding was measured in the absence or in the presence of a 500 µM Biotin-Ahx-PL3 (KJ Ross-Petersen aps) respectively, after 24 h incubation at 25 °C in the dark, sealed with moisture barrier (4Titude, Cat# 4ti-0516/96). The concentration of fluorescent ligand and proteins in-stock solutions was determined by absorbance (for FAM-PL3 ε_495_  =  75000 M^−1^ cm^−1^, for NRP1 ε_280_  =  67630 M^−1^ cm^−1^ and TNC-C ε_280_  =  8480 M^−1^ cm^−1^ were used). The measurements were performed at 25 °C on a Synergy NEO (BioTek) microplate reader using an optical module with an excitation filter at 485 nm (slit 20 nm), emission filter at 528 nm (slit 20 nm) and polarizing beam splitting for dual-channel detection. Dual emission detection mode allows simultaneous recording of intensities that are parallel (I_||_) and perpendicular (I_⊥_) to the plane of excitation light. Sensitivities of channels (G factor) were calibrated with gain adjustment of the photomultiplier tubes using fluorescein (1 µM reference solution, Lambert Instruments) as a standard. The fluorescence anisotropy values were calculated as parameters FA from the equation X: FA = (I_||_−G·I_⊥_)/(I_||_ + 2·I_⊥_). The binding affinity was estimated by global fitting of the data as in^25^. This simultaneous fitting of total and non‐specific binding data takes into account the ligand depletion by both binding processes.

### Nanoparticle synthesis and functionalization

The iron oxide nanoworms (NWs) were prepared according to a published protocol by^8,26,27^. The aminated NWs were PEGylated using maleimide-5K-PEG-NH. Peptides were coupled to NWs through a thioether bond between the thiol group of a cysteine residue added to the N-terminus of the peptide. The concentration of the NWs was determined by measuring the absorbance of NWs at 400 nm with a NanoDrop 2000c spectrophotometer (Thermo Scientific)^8,27^. Silver nanoparticles (AgNPs) were synthesized and functionalized as described^28^, CF647- N-hydroxysuccinimide-dye (NHS-dye) was conjugated to the PEG terminal amine groups, and biotinylated peptides were coated on the NeutrAvidin (NA) on the surface of the AgNPs. Transmission electron microscopy (TEM, Tecnai 10, Philips, Netherlands) was used to image the NPs and DLS (Zetasizer Nano ZS, Malvern Instruments, UK) was used to assess the zeta potential, polydispersity, and size of nanoparticles.

### *In vivo* play-off phage auditioning

*In vivo* play-off was used for internally controlled and competitive systemic phage homing studies in mice bearing tumor xenografts. Phages displaying the candidate TNC-C binding peptides and control peptides were individually amplified and purified by precipitation with PEG-8000 (Sigma-Aldrich, St. Louis, MO, USA), followed by CsCl_2_ gradient ultracentrifugation and dialysis. The phages were pooled at equimolar representation (Table 1 for details) and injected iv at 1 × 10^10^ pfu (in 200 µl PBS) in tumor-bearing mice, circulated for 2 h, followed by anesthesia and intracardial perfusion with DMEM. The tumors and organs were collected in lysogeny broth (LB) medium containing 1% NP40, and the tissues were homogenized using a hand-held homogenizer. Phages in the tissue lysate were amplified in E.coli, purified by precipitation with PEG-8000, and DNA was extracted using a DNA extraction kit (High Pure PCR Template Preparation Kit; Roche, Basel, Switzerland). Next-generation sequencing of phage genomic DNA with Ion Torrent system (Thermo Fisher Scientific, Waltham, MA, USA) was used to evaluate the representation of each peptide-phage in the input mixture, in tumors and control organs. The FASTQ data from Ion Torrent were processed using a custom python script that identified the barcodes, constant flanking residues, and extracted the reads of the correct length.

**Table 1:** *In vivo* play-off audition of TNC-C-selected phages.

Phage-displayed peptides in the“play-off” cocktail			Representation of phage in tumors or in control brain tissue (fold G7 control phage)					
			WT-GBM	P3 SCL	P13	U87-MG	PC3	Norm. brain
Control	GGGGGGG	Control	1.0	1.0	1.0	1.0	1.0	1
TNC-C-selected (round 5)	AGVGRLRRAKLAAALE	Clone-1	2.3	0.9	0.9	1.0	1.4	0.2
	CRGVLRRAKLAAALE	Clone-2	1.6	0.4	0.4	0.7	0.9	0.1
	AVRGRLRVAKLAAALE	Clone-3	1.3	0.6	0.6	0.8	1.0	0.1
	CSRRGILRAKPAAALE	Clone-4	1.5	0.9	0.8	1.1	1.2	0.1
	AGRGRLVRAKLAAALE	Clone-5	23.9	0.4	0.1	0.2	0.3	0.0
	VGRVRFSRKLAAALE	Clone-30	2.5	0.4	0.5	0.7	1.2	0.1
	RRLVRVA	Clone-35	1.4	0.3	0.4	0.6	0.9	0.1
	RGRLVRA	Clone-45	3	0.3	0.4	0.8	1.8	0.1
	GRLTRVR	Clone-46	1.9	0.5	0.5	0.9	0.9	0.1
Clone 5-derivative peptides	**AGRGRLVR**	**Modified clone-5 (PL3)**	**24.1**	**2.1**	**4.7**	**2.1**	**3.9**	**0.4**
	CAGRGRLVRC	Modified clone-5	0.9	0.2	0.4	0.1	0.4	0.0
	RGRLVRAK	Modified clone-5	23.8	0.3	0.1	0.5	3.0	0.2

An equimolar mix of TNC-C-selected phages was i.v. injected in mice bearing WT-GBM, P3 stem cell-like, P13, and U87-MG glioblastoma, or PC3 prostate carcinoma xenografts at 1 × 10^10^ pfu/mouse. After 2 h circulation, background phages were removed by perfusion. Representation of each phage in tumor tissue or in normal brain was assessed by Ion-Torrent high-throughput sequencing. In the tumor tissue, clone 5-derivative phage PL3 (AGRGRLVR) showed the highest representation across tumor models tested. The data represent mean; N = 3 mice for each model.

### *In vivo* fluorescence imaging with the IVIS Spectrum system

PL3 peptide tumor homing in mice bearing U87-MG xenografts was assessed by *in vivo* fluorescence imaging using the IVIS Spectrum imaging system (PerkinElmer, Waltham, MA). AgNPs coated with CF647 dye-labeled neutravidin were functionalized with biotinylated PL3 peptide (PL3-AgNP) or biotin (AgNP). AgNPs were iv injected, and the mice were placed in a dark chamber under isoflurane anesthesia for imaging (parameters: specific excitation filter, 650 nm; emission filter, 665 nm; auto exposure time; binning, medium; the field of view, 12; f/stop, 2). The images were captured at pre-injection (0 h) and 5 h post-injection. The signal was expressed in total Radiant Efficiency [p/s]/[µW/cm²]. The regions of interest (ROIs) were drawn for the whole tumor after tissue background correction, and radiant efficiency signal was quantitated. An automated spectral unmixing algorithm was used, images were analyzed by using Living Image 4.4 software (Caliper Life Sciences, Hopkinton, MA). Three animals per experimental group were analyzed. For the receptor blocking studies, PL3-AgNP injection was preceded by systemic pre-injection of blocking TNC-C and/or NRP1 antibodies (30 µg/mouse) 15 min prior to injection of the AgNPs.

### Cell binding and internalization experiments

U87-MG, PPC1, and M21 cells cultured on glass coverslips were incubated with CF555-labeled AgNPs at 37 °C for 1 h, followed by washing with medium to remove background AgNPs. Etching solution freshly diluted from 0.2 M stock solutions of Na_2_S_2_O_3_ and K_3_Fe(CN)_6_ to 10 mM working concentration in PBS was applied to cells for 3 min, followed by PBS washes. The cells on coverslips were fixed with −20 °C methanol for 1 min. The cell membrane was visualized by staining with Alexa Fluor 488-labeled wheat germ agglutinin (WGA) at 1:1000 at RT for 1 h. Nuclei were stained with 4′,6-diamidino-2-phenylindole (DAPI, Molecular Probes) at 1 µg/mL. Fluoromount-G (Electron Microscopy Sciences) medium was used to mount the coverslips on microscope slides for confocal imaging.

### Tumor-targeted delivery and biodistribution studies

FAM-labeled PL3 peptide coupled NWs or control FAM-NWs (7.5 mg /kg) in PBS were injected into the tail vein of mice bearing subcutaneous U87-MG, PC3, and orthotropic WT-GBM tumors. After 5 h circulation, the mice were perfused with 20 ml PBS/DMEM under deep anesthesia and tumors and control organs were collected. Tissues were *ex vivo* imaged using an Illuminatool Bright Light System LT-9900 (Lightools Research, Encinitas, CA, USA) and snap-frozen. The tissues were cryosectioned at 8–10 µm and mounted on a Superfrost plus slides. The sections were equilibrated at RT and fixed with 4% paraformaldehyde at RT for 20 min, or with methanol at −20 °C for 1 min. The immunostaining was performed with the following primary antibodies; rabbit anti-fluorescein IgG fragment (cat. no. A889, Thermo Fisher Scientific, MA, USA), rat anti-mouse CD31 (BD Biosciences, CA, USA), and in-house prepared CF647 or CF546-labeled single-chain ScFV G11. Secondary antibodies were Alexa 488 goat anti-rabbit IgG, Alexa 647 goat anti-rat IgG, and Alexa 546 goat anti-mouse IgG (all Invitrogen, CA, USA). Nuclei were counterstained with DAPI at 1 μg/ml. The coverslips were mounted on glass slides using Fluoromount-G (Electron Microscopy Sciences, PA, USA), imaged using confocal microscopy (Olympus FV1200MPE, Hamburg, Germany), and analyzed using the FV10 –ASW4.2 viewer, Imaris, and Fiji ImageJ software tools^8,27^.

### Experimental therapy

Tumors were induced by s.c. injection of 8 ×10^6^ U87-MG cells into the right dorsal flank of 18–20-week old male nude mice (weight 38 ± 5 g). When the tumor volume reached 100 ± 20 mm^3^, the mice were assigned randomly into 4 groups (N = 6 per group). For experimental therapy, the mice were treated with i.v. injections of 100 μl of PBS, FAM-_D_[KLAKLAK]_2_-NWs, FAM-PL3-NWs, and FAM-PL3-_D_[KLAKLAK]_2_-NWs every other day for 10 injections. Every 2 days the body weight and animal well-being (behavior, appearance, grooming) were assessed, and the tumor volume was measured using a digital caliper. The tumor volume was calculated with this formula: Volume (V) (mm^3^) = [length × (width)^2^]/2. The study was terminated when the tumor volume reached 2000 mm^3^ (or >20% body weight). At the end of the study, the mice were perfused, followed by tumors, and control organs were excised and snap-frozen for histological studies. Tumor volume, Kaplan–Meier survival and body weight curves were calculated for each group using the GraphPad Prism 6 software with p values <0.05 were considered significant.

### Overlay assay on clinical tumor samples

All methods for the use of human samples were carried out in accordance with relevant guidelines and regulations. The collection and use of human samples was approved by the Research Ethics Committee of the University of Tartu, Estonia (permit #243/T-27). Freshly excised human samples were obtained during surgeries from Department of Neurosurgery, Tartu University Hospital, Estonia. The informed consent was obtained from all patients. The tissue samples were snap-frozen in liquid nitrogen, cryosectioned at 10 μm, fixed with methanol, and permeabilized with PBST buffer (1X Phosphate-Buffered Saline, 0.1% Tween 20), followed by incubation with blocking buffer containing 5% BSA, 5% goat serum, 5% FBS in PBST. For overlay, the sections were incubated with 20 μg/slide PL3-NW and NW overnight at 4 °C. The sections were washed and blocked with a blocking buffer, followed by immunostaining using rabbit anti-fluorescein primary antibodies and detection with the Alexa-488 anti-rabbit and mouse anti-TNC-C antibody and detection with the 647 goat anti-mouse IgG^8^.

### Statistical analysis

Prism 6 software was used for statistical analysis. The results were presented as mean with error bars indicating ±SEM. For a comparison of 2 groups, we used a comparison using an unpaired t-test and multiple groups ANOVA test. P < 0.05 was considered significant and P-values were indicted as follows: *P ≤ 0.05, **P ≤ 0.01, ***P ≤ 0.001 and ****P ≤ 0.0001.

## Results

### Identification of a TNC-C binding peptide

**Figure 1:**
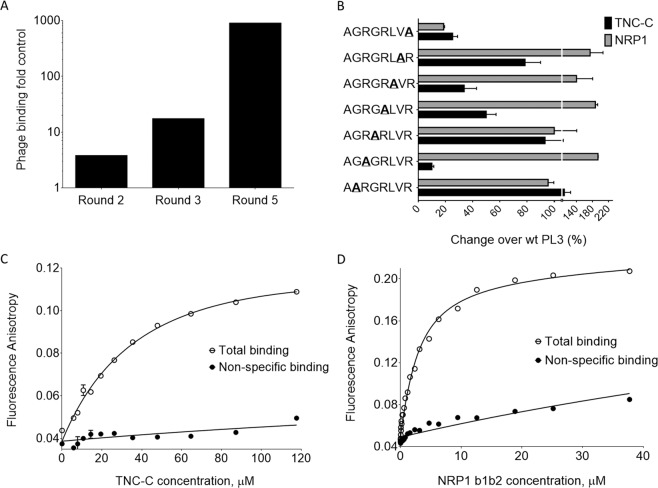
Identification and cell-free binding of PL3 peptide. (**A**) Selection of CX7C T7 phage library on immobilized TNC-C resulted in ~1000-fold increase in phage binding in round 5. Binding is expressed fold heptaglycine (G7) control phage. (**B**) Structural requirements for binding of PL3 peptide to TNC-C and NRP-1. T7-displayed PL3 peptide AGRGRLVR was subjected to alanine scanning mutagenesis (alanine substitutions indicated by underlined bold). Phage binding to TNC-C and NRP-1 is expressed as percent binding of parental PL3 phage. Values represent mean ± standard deviation from 3 independent experiments. (**C,D**) Saturation curve for the binding of FAM-Cys-PL3 to TNC-C and NRP1. FAM-Cys-PL3 (0.66 µM) was incubated with different concentrations of proteins in the absence (total binding, open circles) or presence (nonspecific binding, filled circles) of 0.5 mM Biotin-Ahx-PL3. After a 24 h incubation at 25 °C, FA values were calculated according to FA = (I | | − G·I⊥)/ (I | | + 2·I⊥) and fitted globally. The data represents the mean ± the standard deviation of 3 independent experiments.

TNC-C was expressed in *E*. *coli* and purified using affinity chromatography on Ni-NTA matrix (Fig. S1). For identification of TNC-C–interacting peptides, 5-round biopanning with of T7 CX7C peptide phage libraries was performed, using as the target his-tagged TNC-C coated on polystyrene multiwell plates in rounds 1 and 4, and TNC-C immobilized on Ni-NTA magnetic beads in rounds 2, 3 and 5 (Fig. S2). Selection on TNC-C in multiwell plates was included to avoid enrichment for histidine-containing peptides on the Ni-NTA beads. By round 5, ~1000-fold enrichment in the binding of the selected phage pool to TNC-C was observed (Fig. 1A). Sanger sequencing of a peptide-encoding region of the genome of 38 random phage clones from round 4 demonstrated a shift away from original cyclic CX7C library configuration due to frameshifts in the peptide-encoding region (Table. S1) and overrepresentation of the linear peptides containing the following motifs: RGRLXR (7 repeats), RGRLR (18 repeats), and RLXR (12 repeats)(Table S1). Next, we created a panel of T7 phage clones displaying peptides derived from the phage clone 5 (Table S1) that contains the longest enriched motif, RGRLXR, and characterized their binding to TNC-C (Fig. S3).

For evaluation of systemic tumor homing of the candidate TNC-C-targeting peptides, we used *in vivo* phage play-off auditioning^22^. Tumor-bearing mice were injected i.v. with a phage pool of equally represented candidate TNC-C binding and control phages, followed by circulation, perfusion, and assessment of representation of phage clones in malignant tissue (4 glioma models and 1 prostate cancer model) and in normal brain tissue (Table 1). We found that a T7 clone displaying AGRGRLVR octapeptide was overrepresented in tumor tissue across models tested. In the following studies, we focused on AGRGRLVR peptide that we codenamed PL3.

Fluorescent anisotropy is widely used for solution-based characterization of interaction of small fluorescent ligands with larger partners and is thus well suited for studies of binding of FAM-Cys-PL3 (mW:1.46 kDa) to NRP1 b1b2 domain (mW:37.8 kD) and TNC-C (mW: 12.3 kDa). Binding of FAM-PL3 to both targets was saturable, although at different levels, likely due to different rotational mobility of bound ligand due to the ~3 fold difference in molecular weight of TNC-C and NRP-1. The Kd values were obtained by global fitting data to a binding isotherm, assuming single binding site with ligand depletion^25^. For PL3/NRP-1 interaction, the Kd was 1.1 ± 0.2 μM (Fig. 1D) - similar to published Kd values for the C-terminal arginine-displaying CendR peptide with the b1b2 domain of NRP-1^29^. For PL3/TNC-C, the fluorescent anisotropy assay and calculations yielded Kd of 51 ± 19 μM (Fig. 1C). However, we observed dequenching of FAM signal after tryptic digestion of PL3/TNC-C complex (Fig. S17), suggesting involvement of more than one binding sites and thus, a lower Kd. The detailed mode of interaction of PL3 with TNC-C is a subject for follow-up studies.

### PL3 peptide interacts with recombinant TNC-C and NRP-1 and is taken up by cultured tumor cells

We next studied the binding of alanine-substituted PL3-derivative peptide-phages to determine the amino acids important for peptide binding. The substitution of arginine or leucine residues in PL3 peptide resulted in decreased binding to recombinant TNC-C (Fig. 1B). In contrast, alanine substitutions of glycine and valine had a minimal effect. In contrast to robust interaction with TNC-C, the PL3 phage did not bind to fibronectin EDB-domain, a protein with similar size and negative surface charge as TNC-C (Fig. S4). PL3 peptide includes a C-terminal RXXR motif known to target NRP-1 cell and tissue penetration receptor^23^ overexpressed in most solid tumors. PL-3 phage bound to recombinant b1b2 domain of NRP-1 ~200 fold more than heptaglycine control phage (Fig. 1B). Interaction with NRP-1 was dependent on the presence of C-terminal arginine, as the phage with terminal R > A PL3-derivative peptide showed a dramatically reduced binding (Fig. 1B).

**Figure 2:**
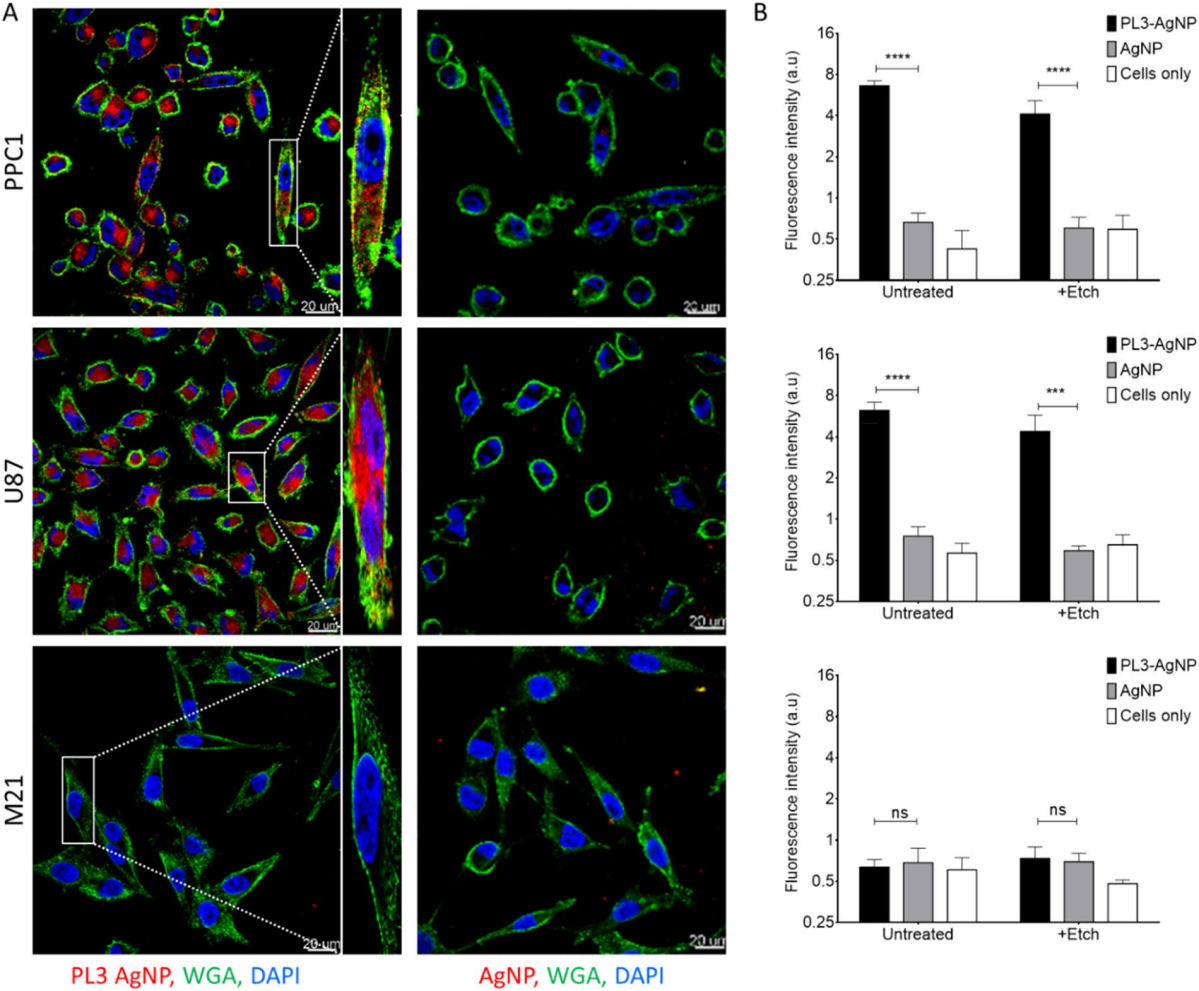
PL3-functionalized AgNPs are taken up in NRP-1-positive PPC1 cells. PL3-AgNPs, or control AgNP particles (37 µL of 100 O.D. stock solution) labeled with CF555 fluorophore were incubated with PPC1 prostate carcinoma, U87-MG glioma, or M21 melanoma cells for 1 h, washed, treated with an optional hexacyanoferrate/thiosulfate redox-based etching solution to dissolve extracellular particles, and processed for confocal imaging. (**A**) Confocal imaging of cells incubated with peptide-targeted vs. control AgNPs. Note robust uptake of PL3-AgNPs (red) in NRP-1-positive PPC1 and U87-MG (and not NRP-1 negative M21) cells (high-magnification images are on the right of PL1-AgNP images). Control particles did not bind to the cells independent of their NRP-1 expression status. Scale bar: 20 µm (main images), and 2 µm (insets). (**B**) Quantitation of binding and internalization of CF555-labeled particles using ImageJ for samples from (**A**) and at least 3 independent experiments were carried out for individual conditions. Error bars: mean ± SD (N = 6), P-values were determined using unpaired Student’s t-test (ns, P > 0.05; ***p ≤ 0.001; ****p < 0.0001).

Many cultured tumor cell lines overexpress NRP-1 and internalize peptides with active CendR motif. We next studied the uptake of CF555-labeled PL3-AgNPs in TNC-C and NRP-1-positive U87-MG glioma cells, NRP-1-positive PPC1 prostate carcinoma cells, and in TNC-C and NRP-1-negative M21 melanoma cells (Fig. S15). The dye-labeled AgNP cores are well suited for fluorescence imaging as the AgNP increases the brightness of the surface-bound dye by about an order of magnitude by plasmonic enhancement. The AgNPs labeled with CF647 dye were coated with neutravidin (NA) and PEGylated as described previously, creating a stable colloid ready for coating with biotinylated peptides. The TEM and DLS showed that particles had an average size of 66.9 ± 27.6 nm, and zeta potential of −5.09 ± 0.19 mV in PBS (Fig. S5). After 1 h incubation with U87-MG and PPC1 cells, PL3-AgNPs were robustly endocytosed with perinuclear accumulation, whereas control nanoparticles showed only a background binding (Fig. 2A,B). In contrast, NRP-1-negative M21 cells showed a low background-like uptake with both PL3 and control AgNPs. Extracellular AgNPs can be removed by treatment with a mild biocompatible hexacyanoferrate/thiosulfate redox-based destain solution so that only internalized AgNP signal remains^30^. In U87-MG and PPC1 cells, etching resulted in a modest decrease of the cellular PL3-AgNP signal (Figs. 2B, S13), suggesting that most of the cell-associated particles were internalized and protected from etching by cellular membrane. The uptake of PL3-AgNPs in U87-MG cells was NRP-1-dependent, as incubation with function-blocking anti-NRP-1 antibody abolished PL3-AgNP signal in U87-MG cells treated with an etching solution to remove extracellular AgNPs (Fig. S14). These experiments showed that PL3 peptide interacts with TNC-C and NRP-1 and that PL3-functionalized nanoparticles are taken up by NRP-1-positive cultured cells.

### PL3-functionalized nanoparticles accumulate in malignant lesions

Systemic *in vivo* phage play-off studies showed that PL3 peptide-displaying phage outperforms other TNC-C binding candidate peptide phages in tumor homing. We next studied the effect of functionalization with synthetic PL3 peptide on *in vivo* tumor tropism of two classes of synthetic nanoparticles, iron oxide NWs and AgNPs.

**Figure 3:**
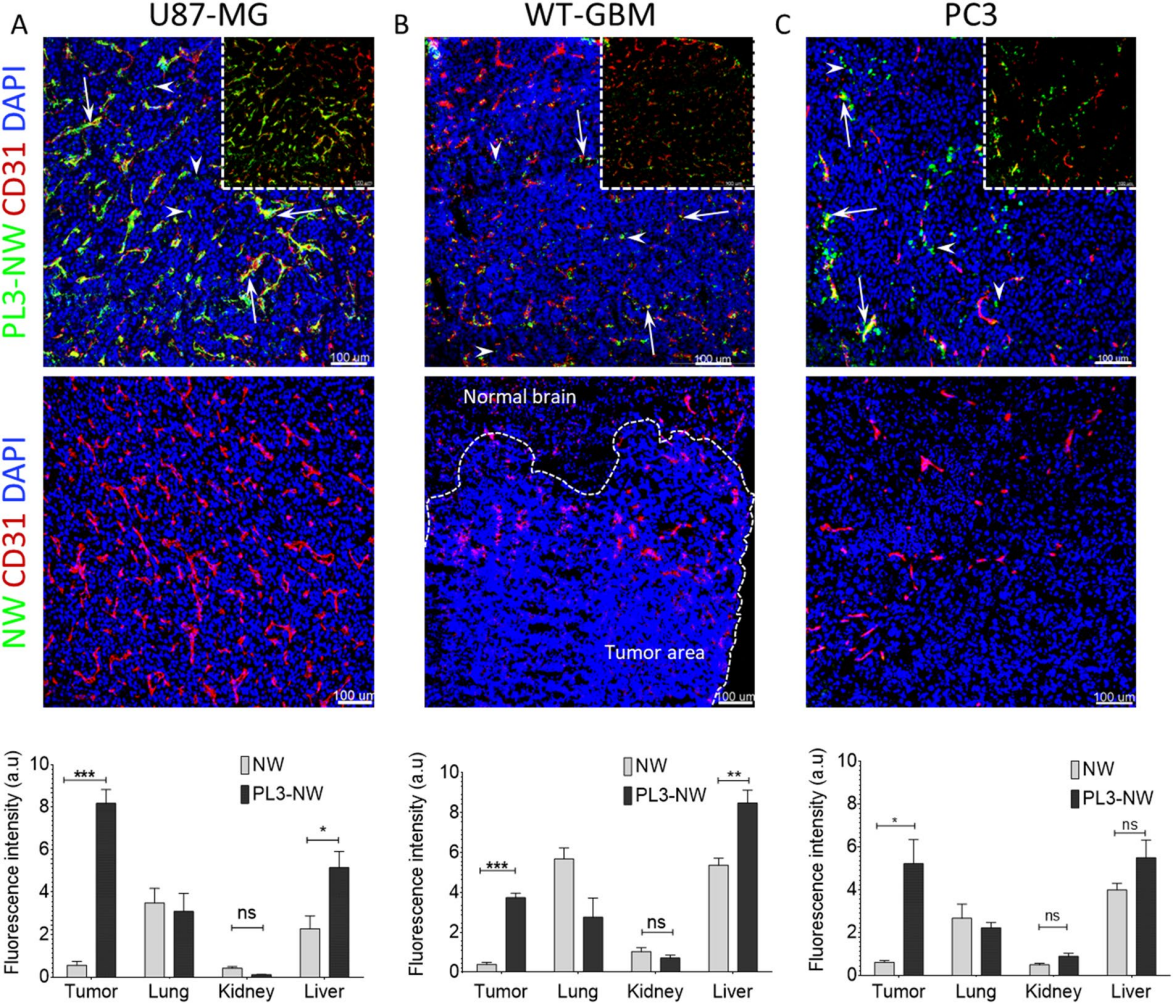
Systemic PL3-NWs accumulate in solid tumors. PL3-NWs or control FAM-NWs were injected i.v. at 7.5 mg/kg into mice bearing s.c. U87-MG glioblastoma (**A**), orthotopic WT-GBM glioblastoma (**B**), or s.c. PC3 prostate carcinoma (**C**). Five h later, the animals were intracardially perfused with DMEM/BSA and the tumors and control organs were snap-frozen, sectioned, immunostained with rabbit anti-FAM (green) and rat anti-CD31 (red) antibodies, counterstained with DAPI (blue) and imaged by confocal microscopy. Arrows point to PL3-NWs in the CD31-positive vessels and arrowheads point to extravasated PL3-NWs in the tumor parenchyma. Insets: confocal images without DAPI channel. Bar charts: quantitation of homing of NWs by quantitative analysis of FAM fluorescence in tissue sections by Fiji ImageJ. Error bars: mean ± SEM (N = 3–6). Scale bars: 100 μm. P-values were calculated using unpaired Student’s t-test (ns p > 0.05; *p < 0.05; **p ≤ 0.01; ***p ≤ 0.001).

**Figure 4:**
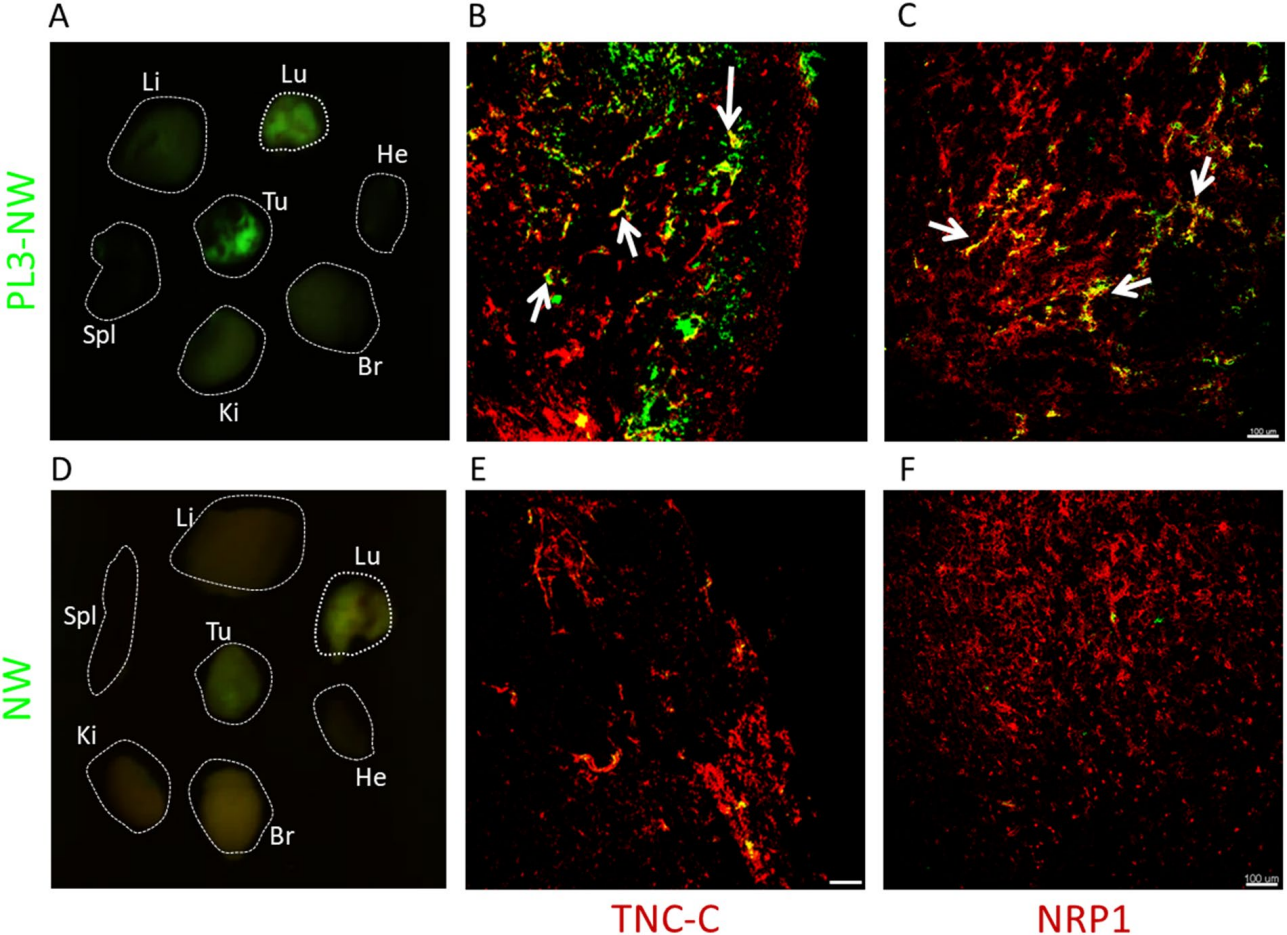
PL3-NWs in tumor localize at areas positive for TNC-C and NRP1 immunoreactivities. PL3-NWs (**A–C**, upper row), or control NWs (**D–F**, lower row) were i.v.-administered at 7.5 mg/kg into mice bearing s.c. U87-MG glioblastoma. Five h later, the animals were intracardially perfused with DMEM, and tissues were collected for *ex vivo* macroscopic imaging and fluorescence microscopy. (**A,D**) *Ex vivo* macroscopic Illumatool imaging of PL3 and control NWs in green channel. Tu: tumor; Br: brain, Ki: kidney; Spl: spleen; Li: liver; Lu: lung; He: heart. The images are representative of 3 independent experiments. (**B,C,E,F**) Confocal imaging of NWs, TNC-C, and NRP-1 in tumor cryosections. PL3-NWs colocalize with perivascular TNC-C and NRP1 (arrows), whereas FAM-NWs show only background accumulation. Tumor tissues were stained with rabbit anti-FAM (green), anti-TNC-C ScFv G11 (red), and rabbit anti-NRP1 (red) antibodies. Arrows point to colocalization of PL3-NW with TNC-C and NRP1. Scale bar, 100 µm. The images are representative of 3 independent experiments.

We first tested the effect of PL3 functionalization on tumor homing of dextran-coated PEGylated paramagnetic NWs – a dual-use model nanoplatform that can be used as a carrier for drugs and for MR imaging due to intrinsic T2 contrast properties^8,26^. NWs with an average size of 88.8 ± 5 nm and zeta potential of −7.8 ± 2 mV (Fig. S6) were coated with FAM-labeled PL3 peptide or FAM-Cys control with no significant effect on particle size or surface charge. NWs were i.v. administered at 7.5 mg/kg NWs in mice bearing prostate cancer xenografts (PC3 s.c. tumors), or gliomas (s.c. U87-MG and orthotopic WT-GBM), both known to overexpress TNC-C and NRP-1^8,27^. After 5 h circulation, accumulation of PL3-NWs, but not control NWs, was observed in all 3 tumor models tested (Fig. 3A–C). Confocal analysis demonstrated that whereas the PL3-NW homing was mainly overlapping or associated with CD31-positive vascular structures (Fig. 3A–C, arrows), in some regions, the PL3-NWs extravasated and accumulated in the tumor parenchyma (Fig. 3A–C, arrowheads). In control organs, PL3-NWs and control NWs showed similar background (Fig. S7). Macroscopic imaging confirmed preferential tumor accumulation of the PL3-NWs in U87-MG tumor mice (Fig. 4A,D). In tumor tissue PL3-NWs were found in the areas positive for TNC-C and NRP-1 immunoreactivities (Fig. 4B,C,E,F).

We also studied the binding of PL3 and control NWs to the clinical glioma. The NWs were overlaid on cryosections of GBM, washed, and subjected to confocal imaging. PL3-NWs showed co-localization with TNC-C-positive structures in tumor perivascular space and parenchyma (Figs. S8,9). For TNC-C detection we used an in-house monoclonal antibody, and as a specificity

**Figure 5:**
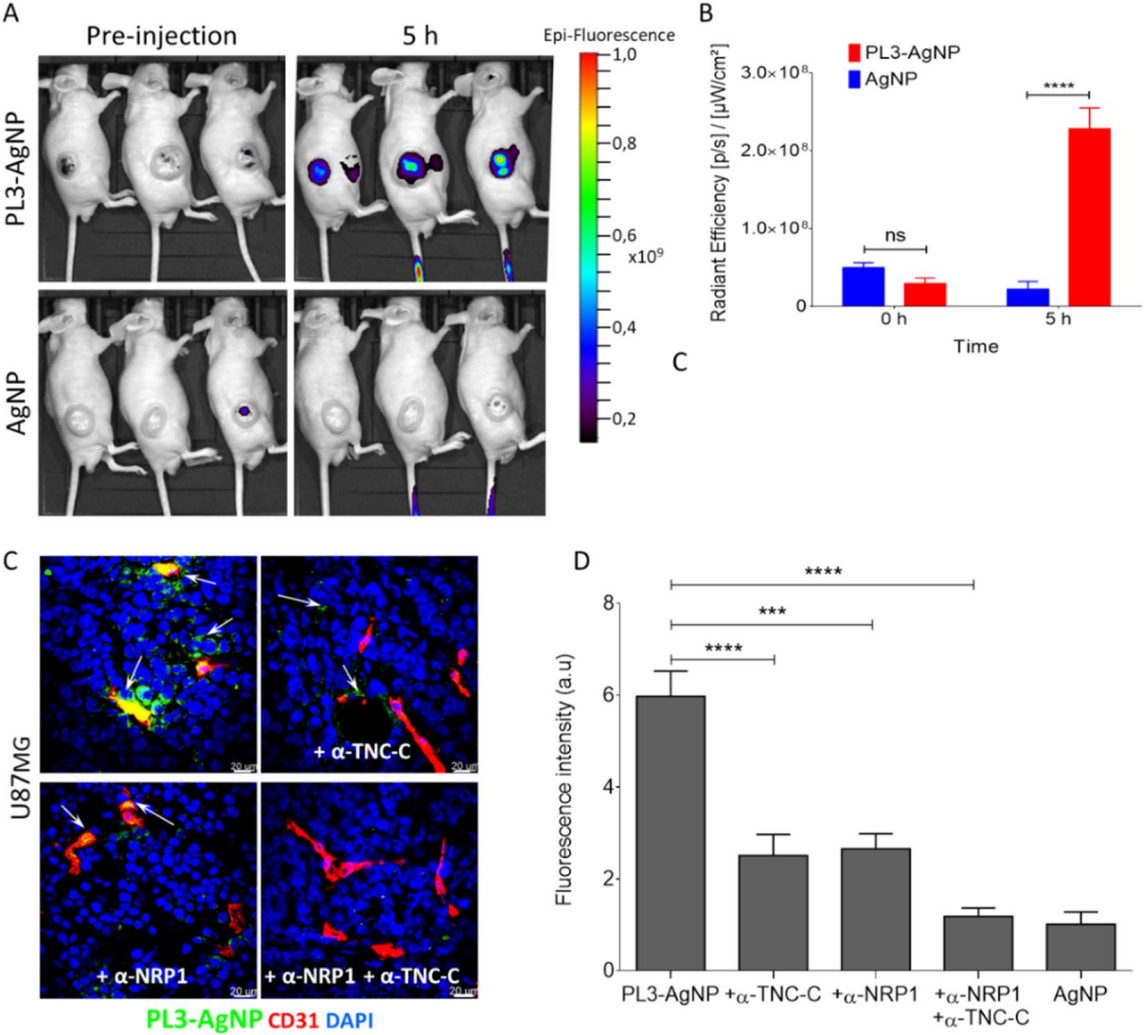
Systemic PL3-AgNPs home to U87-MG tumors. (**A**) *In vivo* imaging of U87-MG s.c. tumor-bearing mice injected with Alexa-647-labeled PL3-AgNPs (upper row), or with non-targeted AgNPs (lower row). The fluorescence imaging was performed using IVIS Lumina Imaging System at pre-injection and at 5 h post-injection. Three mice per group were i.v. injected with AgNPs; images after spectral un-mixing are shown. Note elevated tumor Alexa-647 signal in mice injected with PL3-AgNPs at 5 h post-injection. (**B**) Quantification of tumor Alexa-647AgNP fluorescence at pre-injection and at 5 h time points. The signal is expressed as Average Radiant Efficiency [p/s]/[µW/cm²]. Error bars: mean ± SEM (N = 3); P-values were determined using two-way ANOVA Fisher’s LSD test (ns, P > 0.05; ****P ≤ 0.0001). (**C**) The PL3-AgNP green signal was quantified from representative images using Fiji ImageJ. Error bars, mean ± SEM (N = 3 mice per group); scale bars: 20 μm; p-value was determined using Student unpaired t-test, two-tailed; ***p ≤ 0.001; ****p < 0.0001. (**D**) Effect of TNC-C and NRP-1 antibodies on tumor accumulation of PL3-AgNPs. PL3-AgNPs alone, or in combination with anti-TNC-C or anti-NRP1 antibodies, or a cocktail of both antibodies were i.v injected into mice bearing U87-MG xenograft tumors. Mice were perfused through the heart with PBS/DMEM 5 h after injection and organs were collected for cryosectioning and confocal microscopy. Colors: PL3-AgNPs (green), CD31-positive blood vessels (red) and DAPI (blue).control, we confirmed that preincubation of the antibody with recombinant TNC-C resulted in reduced staining (Fig. S10).

We also tested the effect of PL3 coating on tumor homing of near-infrared dye-labeled AgNPs. Intravital imaging with IVIS Spectrum after 5 h circulation showed that the PL3-CF647-AgNPs accumulated in U87-MG lesions at ~10 fold more than control CF647-AgNPs (Fig. 5A,B). Tumor homing of PL3-AgNPs was confirmed by confocal imaging (Fig. 5C). Coadministration of PL3-AgNPs with blocking rabbit polyclonal antibodies against either TNC-C or NRP-1 resulted in a decrease in tumor homing, and a cocktail of both antibodies almost completely inhibited the tumor accumulation (Fig. 5C,D). These data show that PL3 functionalization results in specific tumor homing of different classes of nanoparticles.

### PL3-guided proapoptotic NWs have anti-glioma activity

**Figure 6:**
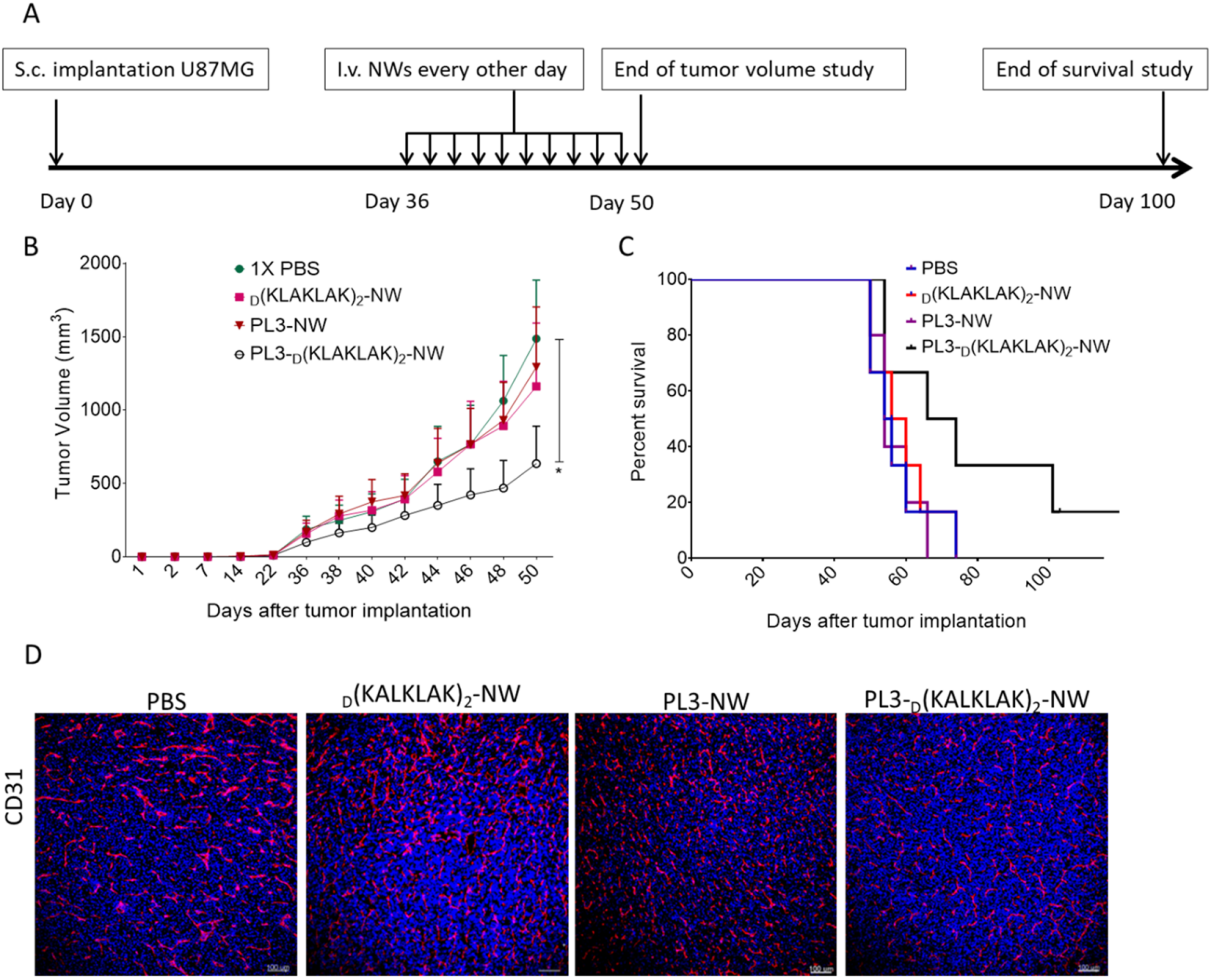
Experimental therapy with PL3-_D_(KLAKLAK)_2_-NWs suppresses glioblastoma growth. (**A**) Experimental design of the tumor treatment study. Treatment of mice bearing s.c. U87-MG tumors with systemic injections of NWs were initiated on day 36 when the tumors had reached 100 mm^3^ volume; groups of 6 randomized mice were treated every other day for 10 injections. The body weight, survival, behavior, and tumor volume were monitored every two days until the end of the treatment. (**B**) Tumor size dynamics of mice treated with 5 mg/kg of _D_(KLAKLAK)_2_-NW, PL3-NW and PL3-_D_(KLAKLAK)_2_-NW, or control PBS (N = 6 mice/group). The tumor volume was measured with a digital caliper and calculated using the formula: Volume (V) (mm^3^) = [length × (width × 2)]/2. Data were analyzed with 2-way ANOVA and log-rank test. Error bars: mean ± SEM; *P < 0.05. (**C**) Kaplan-Meier survival analysis. At the endpoint of the study the mice were sacrificed by perfusion, and organs and tumors were collected. Tumor volume, Kaplan–Meier survival curve and body weight curve were calculated for each group using the GraphPad Prism 6 software with p values <0.05 considered significant. (**D**) Treatment with PL3-_D_(KLAKLAK)_2_-NWs resulted in decreased tumor vascularization. Cryosections of tumors were stained with vascular endothelial marker CD31 to visualize tumor blood vessels using confocal microscopy. Red: blood vessels stained by the anti-CD31 antibody; blue: nuclei stained with DAPI. The images are representative of 3 independent tumors.

We next tested the effect of PL3-functionalization on anticancer efficacy of nanoparticles loaded with proapoptotic _D_(KLAKLAK)_2_ peptide. For these studies we used s.c. U87-MG tumor model in order to monitor tumor size, rather than survival, as the endpoint. Starting on day 36 after tumor induction (when the tumors had reached 100 mm^3^), the tumor mice were treated with i.v. PL3-_D_(KLAKLAK)_2_-NWs, _D_(KLAKLAK)_2_-NWs, PL3-NWs, or PBS for 10 injections every other day, and tumor volume and survival of the mice were recorded (Fig. 6A). Over the treatment, the volume of tumors in PBS, PL3-NW, and _D_(KLAKLAK)_2_ –NW-treated mice increased rapidly. In contrast, tumor growth in the _D_(KLAKLAK)_2_ -PL3-treated group was significantly delayed (Fig. 6B). The median survival of PBS, _D_(KLAKLAK)_2_-NW, PL3-NW, and PL3-_D_(KLAKLAK)_2_-NW groups was 55, 58, 54, and 70 days, respectively. In the _D_(KLAKLAK)_2_ -PL3 group, 50% of mice had prolonged survival compared to the animals in the other treatment groups (Figs. 6C and S11). Immunostaining of post-treatment tumor tissue with anti-CD31 antibody to visualize tumor vasculature showed that compared to controls PL3-_D_(KLAKLAK)_2_-NW-treated tumors had a significant reduction in CD31-positive area and cleaved Caspase-3 showed a significant increase in apoptotic cells (Figs. 6D, S12, S15).

## Discussion

Most affinity-based precision delivery strategies target receptors on the surface of tumor cells. This approach, while clearly proven useful, has limitations, as it relies on targeting a limiting number of systemically accessible receptors on genetically unstable malignant cells^31^. Compared to surface antigens on tumor cells, tumor-associated ECM represents an abundant and stable target with low shedding^32^. ECM directed affinity targeting strategies may allow simultaneous targeting of both malignant tumor cells and tumor support cells (fibroblasts, immune- and vascular cells) and be beneficial for improving treatment efficacies. TNC, an ECM component expressed at the invasive tumor front and in the angiomatrix, is predictive of adverse outcomes^13^ and provides specific targeting opportunities due to precisely controlled expression of its multiple structurally and functionally different isoforms^4,11^. Here, we report the development of an affinity ligand, octameric PL3 peptide, that targets C-domain of TNC and also interacts with NRP-1, a pleiotropic hub receptor upregulated in angiogenic sites and in malignant tissues involved in regulation of vascular permeability. Systemic PL3 phage nanoparticles and two types of synthetic PL3-guided nanocarriers home to solid tumors implanted in mice and bind to receptor-positive regions in clinical tumor samples. For therapeutic nanoparticles, PL3 functionalization improves their anticancer activity. Our study suggests potential uses for PL3 guided compounds and particles for improved detection and therapy of solid tumors.

Over the years, several preclinical and clinical-stage affinity ligands for TNC have been developed, such as a aptamers^33^, FHK peptide^7^, a bispecific PL1 peptide that in addition to TNC-C also targets fibronectin extra domain B^8^, TNC-C-targeting single-chain G11 antibody^34^, and TNC-C/D targeting monoclonal antibody 81C6^35^. These and other ECM-reactive affinity ligands have proven useful for tumor delivery of extracellularly-acting anticancer payloads such as cytokines/growth factors, or payloads with intrinsic internalizing ability, such as proapoptotic _D_(KLKLAK)_2_ peptide nanoparticles, or cell-permeable cytotoxic compounds^3,32,36^. However, a challenge for these and other ECM-directed systemic compounds is that they can only reach extravascular tumor tissue passively, through the increased leakiness of aberrant tumor microvasculature, a phenomenon known as enhanced permeability and retention (EPR) effect^37^. EPR shows extensive inter- and intratumoral variability, thus decreasing the utility of the EPR-based targeting strategies^38^. During systemic *in vivo* play-off experiments using a panel of phage clones derived from screen on recombinant TNC-C in tumor mice, we observed that a phage clone displaying PL3 peptide with C-terminal RLVR motif outperformed other phage clones that had shown better TNC-C binding under cell-free conditions. C-terminal RLVR of PL3 corresponds to RXXR CendR consensus motif that, when exposed at the C-terminus, interacts with b1 domain of NRP-1 to trigger a trans-tissue pathway that mediates exit from the blood vessels and extravascular transport through tumor tissue^23,39^. Indeed, we observed specific binding of PL3 nanoparticles to recombinant NRP-1 b1b2 domain and not to b1b2 with mutated CendR binding pocket, and binding and internalization of the PL3 nanoparticles in NRP-1-positive PPC1 cells and no interaction with NRP-1-negative M21 melanoma cells. *In vivo*, NPs, including biological bacteriophage nanoparticles, are because of their size particularly prone to be excluded from difficult-to-access parts of tumors, and PL3 functionalization may mitigate this problem. PL3 peptide functionalization results in a small reproducible increase in uptake of NWs in the liver. The liver, along with other organs of the reticuloendothelial system, plays a central role in the nonselective clearance of NPs, primarily due to scavenger and phagocytic functions of liver Kupffer cells^40,41^. PL3-dependent increase in liver accumulation may be due to NRP-1 binding-ability of the peptide, as NRP1 has been detected in hepatic stellate cells and liver sinusoidal endothelial cells^42,43^. A potential strategy to mitigate NRP-1-dependent accumulation of PL3-targeted compounds in nontarget organs may be to render its CendR motif proteolytically activatable, as we have done with urokinase dependent uCendR3 peptide^44^. Numerous studies document the ability of CendR peptides to specifically increase the accumulation of a variety of anticancer therapeutics, such as chemotherapeutic agents, antibodies and NPs, in tumors^45–51^. For PL3 peptide, combination of CendR activity with the tumor ECM homing function may increase accessibility of TNC-C deeper in tumor parenchyma than would be possible under the conditions of physiological EPR with simple docking-based affinity targeting. In 2016, a chimeric 19-mer chimeric peptide composed of TNC-A-D domain binding peptide and tLyP-1 tumor penetrating peptide^52^ that targets NRP-1 on tumor cells was reported to allow anti-glioma drug delivery via NRP-1- and TNC-mediated specific penetration of nanoparticles into glioma parenchyma^53^. Compared to that peptide, the 8-amino acid PL3 peptide we have identified has the advantage of being smaller, and hence less likely to be immunogenic and simpler to produce and develop towards clinical applications. In both these peptides, the CendR motif is C-terminally exposed and does not need on-site proteolytic activation, as seen for several other tumor penetrating peptides. NRP-1 is expressed in the endothelial cells in non-malignant sites, albeit at lower levels than observed in tumors, and systemic peptides with C-terminally exposed CendR motif show increased background, in particular in first-met vascular beds, lung, and heart^23,52^. We have reported on development of tumor penetrating peptides containing cryptic CendR motifs that are cleavage-activated by a tumor-associated serine protease, urokinase-type plasminogen activator for tumor-specific extravasation and tissue penetration^44,54^. Our ongoing studies will explore homing and activation of PL3-derived cryptic CendR peptides with added C-terminal residues compatible with CendR motif activation of the peptide in tumor microenvironment. We hypothesize that this strategy may add additional layer of tumor selectivity to further improve the tumor homing/background ratio for PL3 and other ECM-targeted peptides.

The mode of interaction of PL3 peptide with TNC-C remains to be determined in follow-up studies. At neutral pH, the net charge of PL3 peptide is +3, whereas TNC-C is negatively charged (−5). Whereas electrostatic interactions may contribute to peptide-receptor interaction, the binding is likely to involve other mechanisms also, as no binding of PL3 phage was observed to Fn-EDB (charge −10 at pH 7). Ongoing *in silico* modeling combined with site-directed mutagenesis studies will map the binding site of PL3 on TNC-C. This information can be used to develop PL3 derivatives containing non-natural amino acids or low molecular weight peptidomimetic compounds of improved binding and plasma stability.

In summary, this study describes the identification of an 8-amino acid homing peptide, PL3, that interacts with TNC-C and with the cell- and tissue penetration receptor NRP-1. Systemic PL3-guided nanoparticles accumulated in tumor xenografts implanted in mice. The PL3-guided nanoparticles were useful for tumor detection, imaging and served as a tumor-seeking carrier for a proapoptotic peptide anticancer payload. Our study warrants future studies to study applications for PL3-targeted compounds and nanoparticles for improved detection and therapy of solid tumors.

## Supporting Information

Supporting Information.
