# Supplementary material for: Tumor-penetrating peptide for systemic targeting of Tenascin-C
Source: Sci Rep. 2020 Apr 2;10:5809. doi: 10.1038/s41598-020-62760-y (PMC7118115; doi:10.1038/s41598-020-62760-y)
Supplement: Supplementary file 1 — Supporting Information. [file 41598_2020_62760_MOESM1_ESM.pdf]

Supplementary Information

Tumor-penetrating peptide for systemic targeting of Tenascin-C.

Prakash Lingasamy[1], Allan Tobi[1], Kaarel Kurm [1], Sergei Kopanchuk [7], Aleksander Sudakov [1,6], Markko Salumäe [1], Tõnu Rätsep[4], Toomas Asser[4], Rolf Bjerkvig[5], Tambet Teesalu[1,2,3]†

[1] Laboratory of Cancer Biology, Institute of Biomedicine and Translational Medicine, University of Tartu, Tartu, Estonia.

[2] Cancer Research Center, Sanford Burnham Prebys Medical Discovery Institute, La Jolla, California, USA.

[3] Center for Nanomedicine and Department of Cell, Molecular and Developmental Biology, University of California, Santa Barbara, Santa Barbara, California, USA.

[4] Department of Neurosurgery, Tartu University Hospital, Estonia.

[5] Department of Biomedicine Translational Cancer Research, University of Bergen, Norway.

[6] Oxford Nanopore Technologies Ltd., Oxford, UK.

[7] Institute of Chemistry, University of Tartu, Ravila 14, Tartu, 50411, Estonia.

†Corresponding author:

Tambet Teesalu PhD

Laboratory of Cancer Biology, Institute of Biomedicine and Translational Medicine, University of Tartu, Ravila 14b, Tartu 50411, Estonia. Phone: 372-5397-4441; Fax: 372-737-4268; E-mail: tambet.teesalu@ut.ee

The file includes:

Table S1. T7-displayed peptides from round 4 in vitro biopanning on TNC-C.

Figure. S1. Expression and purification of TNC-C.

Figure. S2. Scheme of biopanning to identify TNC-C-targeting peptides.

Figure. S3. Binding of clone 5 derivative peptide phages to TNC-C.

Fig. S4. PL3 peptide phages binding to TNC-C and NRP1.

Figure. S5. Characterization of AgNPs.

Figure. S6. Characterization of NWs.

Figure. S7. The biodistribution of PL3-NWs in healthy organs.

Figure. S8. PL3-NWs bind to surgical explants of human glioma tissues 1.

Figure. S9. PL3-NWs bind to surgical explants of human glioma tissues 2.

Figure. S10. Anti TNC-C antibody validation on surgical explants of human glioma tissues.

Figure. S11. Experimental tumor therapy with PL3-guided NWs extends survival of glioblastoma.

Fig. S12. Analysis of CD31 immunofluorescence signal in post-treatment tumors by quantitative analysis of FAM fluorescence in a series of images by Fiji ImageJ.

Fig. S13. NRP-1-dependent internalization of PL3-functionalized AgNPs

Fig. S14. NRP-1-dependent internalization of PL3-functionalized AgNPs in U87-MG cells.

Fig. S15. Analysis of cleaved Caspase-3 immunofluorescence signal in post-treatment tumors.

Fig. S16. TNC-C and NRP1 receptor expression in U87-MG, PPC1, and M21 cells.

Fig. S17. Dequenching of FAM-PL3 upon tryptic digestion of FAM-PL3/TNC-C complex.

Supplementary Materials and Methods

Cloning, expression, and purification of TNC-C

Vector Construction. A pF1K (Promega, # FXC00319) derivative plasmid containing full-length cDNA of TNC was used for PCR amplification of cDNA region of TNC-C with Phusion Hot Start II High-Fidelity DNA Polymerase (Thermo Fisher Scientific Inc # F-537L; primer pairs: 5′- CTCCTCTCATATGGAGGCCCTGCCCCTTC -3′ and 5′-CAGACACTCGAGTTATCATGTAACAATCTC -3′ for domain TNC-C. NdeI and XhoI restriction sites underlined). The fragments were cloned in the pET28a+ plasmid for TNC-C expression as an N-terminally His-tagged protein.

Expression and purification of TNC-C. The pET28a+TNC-C plasmid was used for transformation of *E. coli* BL21 Rosetta 2 (DE3) pLysS cells (Novagen, #70956). The protein expression was induced by addition of isopropyl β-D-1-thiogalactopyranoside (IPTG) (Sigma, # I6758) to 0.5 mM final concentration and the bacteria were cultured at 18°C for 16h. Cells were

collected by centrifugation, resuspended in ice-cold IMAC buffer (25mM Tris-HCl, 400mM NaCl, 25mM imidazole pH 8, containing EDTA-free protease inhibitor cocktail and DNase I) and lysed by sonification (Bandelin Sonopuls HD 2070, Germany). The cleared bacterial lysate was purified using HiTrap IMAC HP columns (GE Healthcare # 17-0920-05) on ÄKTA purification system (GE Healthcare), and the eluate was dialyzed against PBS using 3.5 kDa cut-off 3mL Slide-A-Lyzer Dialysis Cassettes (Thermo Scientific #66330). TNC-C concentration was determined by bicinchoninic acid assay (Thermo Scientific #23227), and the purity of the proteins was assessed by SDS-PAGE. Mass spectrometry and *de novo* peptide sequencing were used to confirm the size and sequence of the purified proteins. His-tagged NRP-1 b1b2 domain was expressed and purified as described(Teesalu et al., 2009).

Antibody production

Single-chain antibodies: The cDNA sequences encoding TNC-C-G11-scFV were retrieved from US patent application EP2157102 A1, and synthetic DNA fragments were cloned in the pET28a+ expression plasmid. Recombinant antibodies were expressed in *E. coli* BL21 Rosetta 2 (DE3) pLysS (Novagen, #70956) cells and purified using Protein A GraviTrap Sepharose (GE Healthcare # 28-9852-54), followed by affinity purification on immobilized TNC-C. The purified antibodies were analyzed by SDS-PAGE. Pull-down assays and ELISA were used to verify the interaction of the purified antibodies with the target TNC-C domain.

Clone No.	Peptide sequence	Repeats	TNC-C binding (fold G7 control phage)
1	AGVG**RLRR**AKLAAALE	1	3259
2	CRGVLRRAKLAAALE	4	5955
3	AVRGRLRVAKLAAALE	7	5333
4	CSRRGILRAKPAAALE	5	4889
5	AG*RG**RLVR***AKLAAALE	1	5785
7	AVRGRLRVAKLAAALE	7	7111
35	R**RLVR**VA	2	1223
30	VGRVRFSRKLAAALE	1	3702
36	CQRMGVVGAKLAAALE	2	3928
44	*RG**RLRR***VE	4	886
45	*RG**RLVR***A	2	737
46	G**RLTR**VR	2	115

Table S1. T7-displayed peptides from round 4 in vitro biopanning on TNC-C. Random phage clones from round 4 of selection on TNC-C were subjected to Sanger sequencing of the peptide-encoding segment of the genome. The table shows peptide sequences of the 38 sequenced clones, and quantitation of binding of individual peptide phages to TNC-C (fold binding of control heptaglycine phage). RGRLXR motif (7 total repeats) is shown in italic, RGRLR motif (18) is underlined, and RLXR motif (12) is indicated in bold.

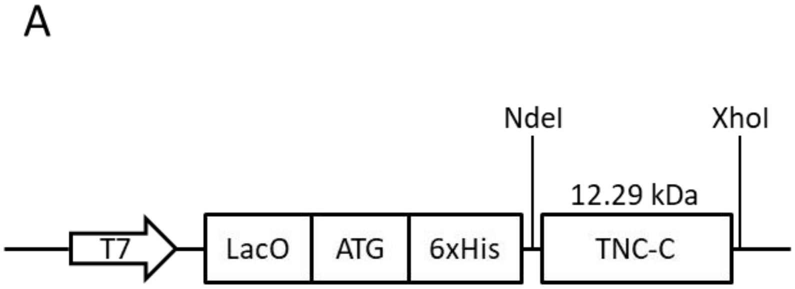

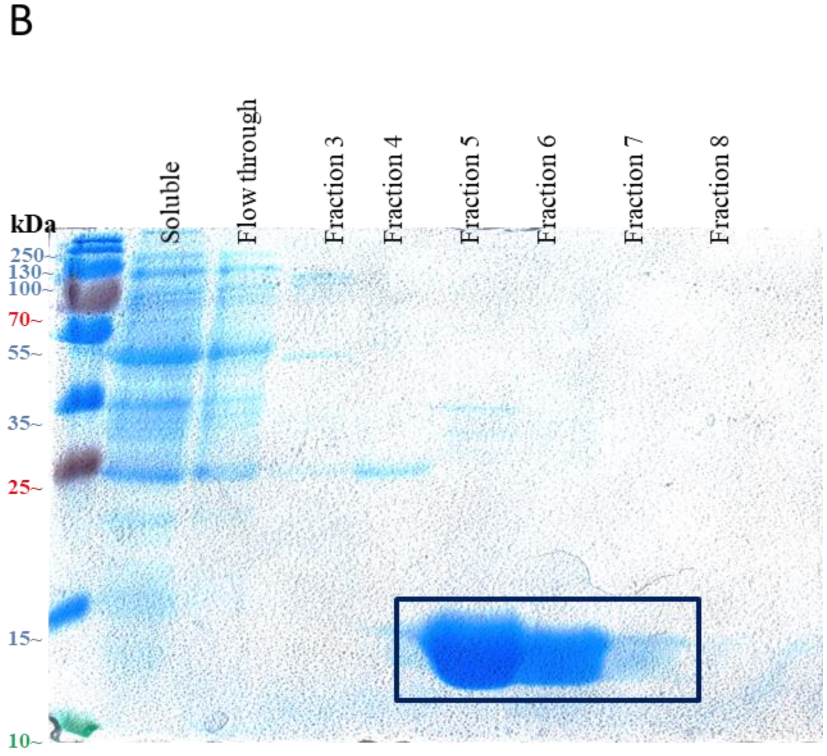

Figure. S1. Expression and purification of TNC-C. (A) Schematic representation of TNC-C bacterial expression cassette in the pET28a+ plasmid. (B) The SDS-PAGE of purified TNC-C highlighted in the box.

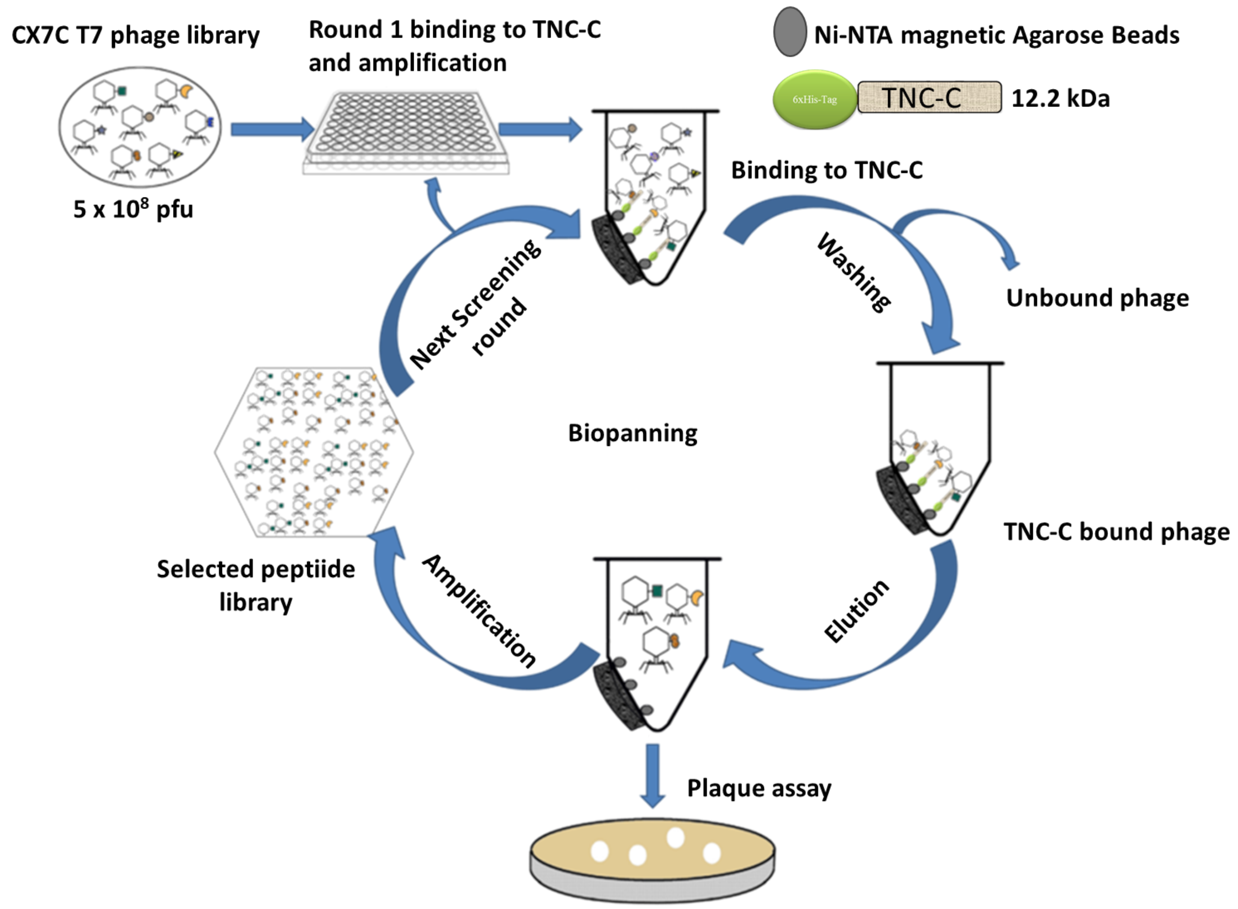

Figure. S2. Scheme of biopanning to identify TNC-C-targeting peptides. CX7C peptide libraries displayed on T7 bacteriophage particles were subjected to a series of selection rounds on recombinant TNC-C. The first and fourth round of screening was performed on TNC-C immobilized on ELISA plates, and following rounds on TNC-C coated on Ni-NTA agarose beads.

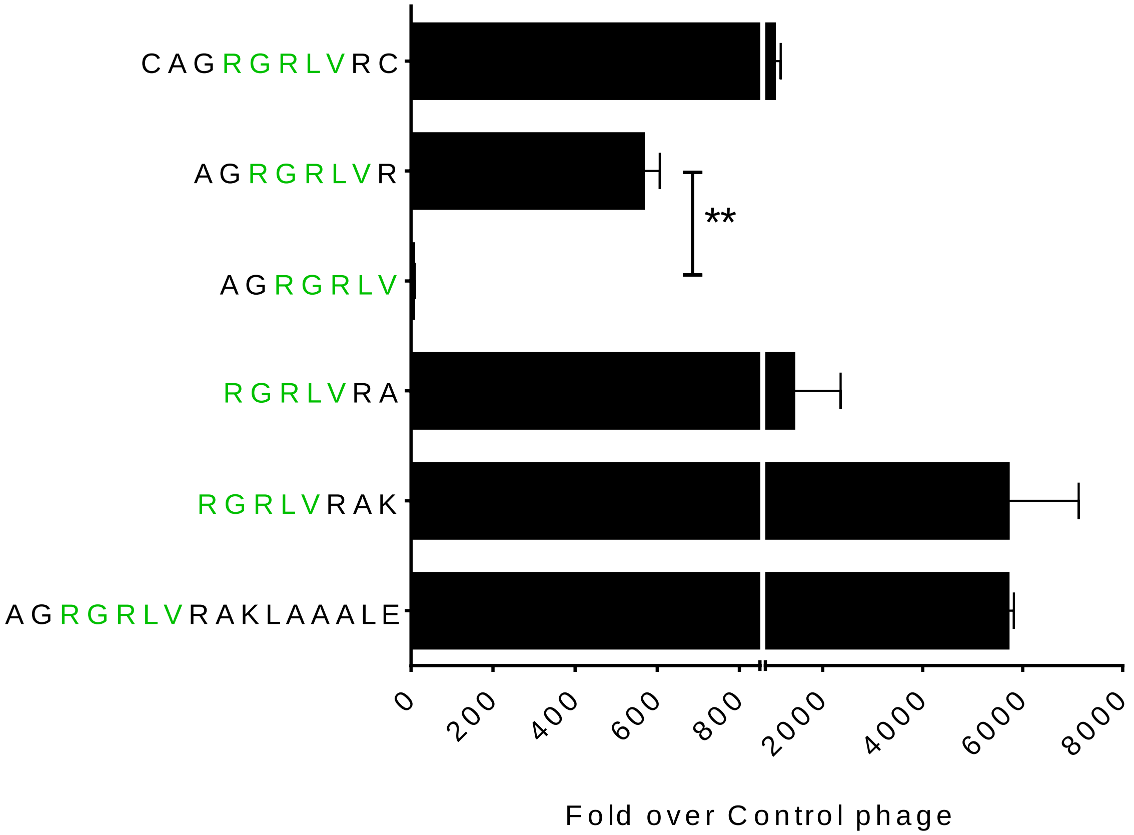

Figure. S3. Binding of clone 5 derivative peptide phages to TNC-C. Binding of individual peptide phages to TNC-C immobilized on Ni-NTA magnetic beads (fold binding of control heptaglycine phage). Error bars: mean ± SEM (N=2). Scale bars: 100 µm. P-values were determined using unpaired Student's t-test (**$p \leq 0.01$).

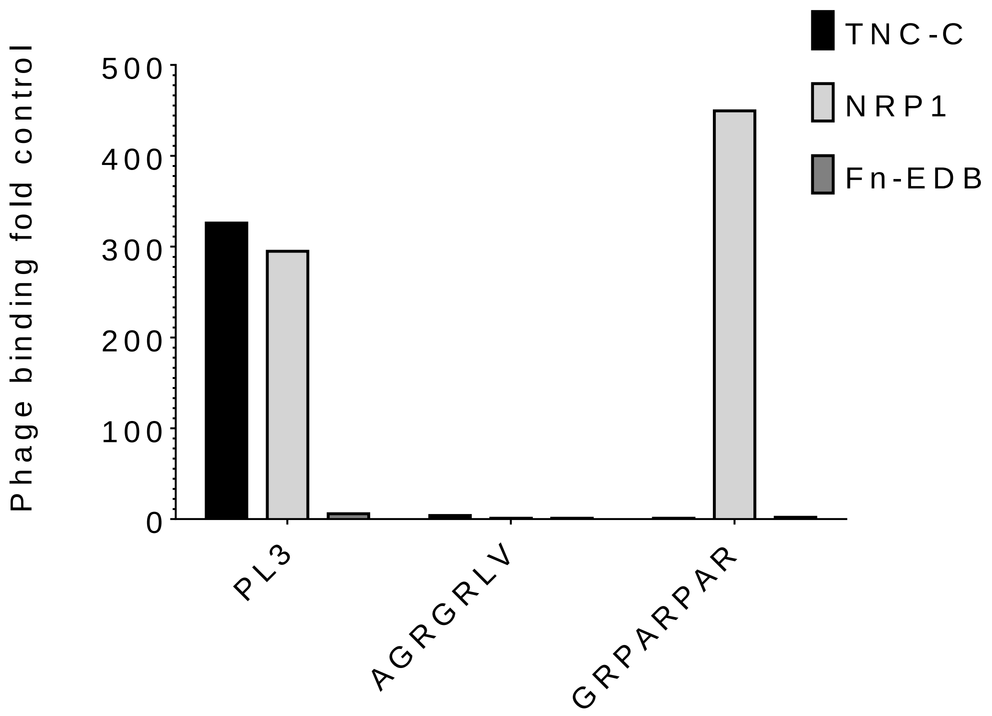

Fig. S4. PL3 peptide phages binding to TNC-C and NRP1. The selected peptide PL3- phage binds to immobilized TNC and NRP1 b1b2, but not to a control protein FN EDB. Phage binding is expressed fold over control phage displaying heptaglycine (G7) peptide. NRP1 binding peptide RPARPAR used as a positive control.

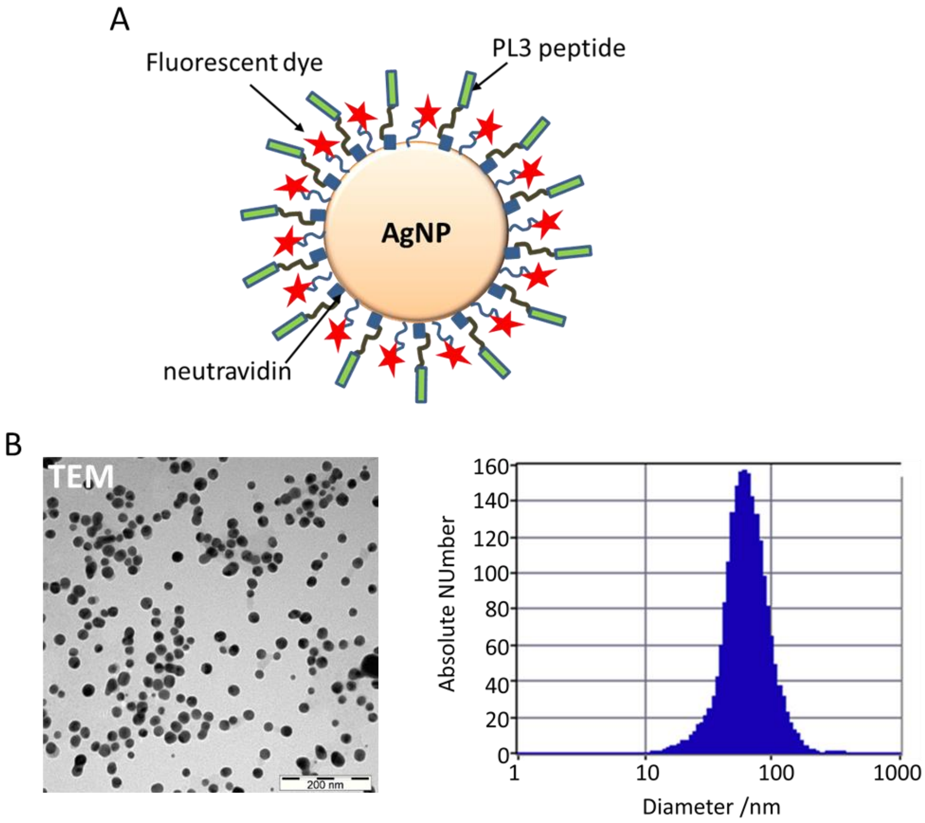

Zeta Potential @25°C: -5.09±0.19mV   Mean size :66.9 ± 27.6 nm

Figure. S5. Characterization of AgNPs. AgNPs were synthesized and functionalized with NeutrAvidin as described (Willmore et al., 2016), CF647 or CF555 N-hydroxysuccinimide-dye (NHS-dye) was conjugated to the terminal amine group of PEG, and biotinylated peptides were coated on the NeutrAvidin (NA) on the surface of the AgNPs. (A) Schematic representation of PL3-AgNPs. (B) Transmission electron microscopy (TEM, Tecnai 10, Philips, Netherlands) was used to image and Dynamic Light Scattering (DLS, Zetasizer Nano ZS, Malvern Instruments, UK) was used to assess the zeta potential, polydispersity, and size of NPs. Scale bar: 200 nm.

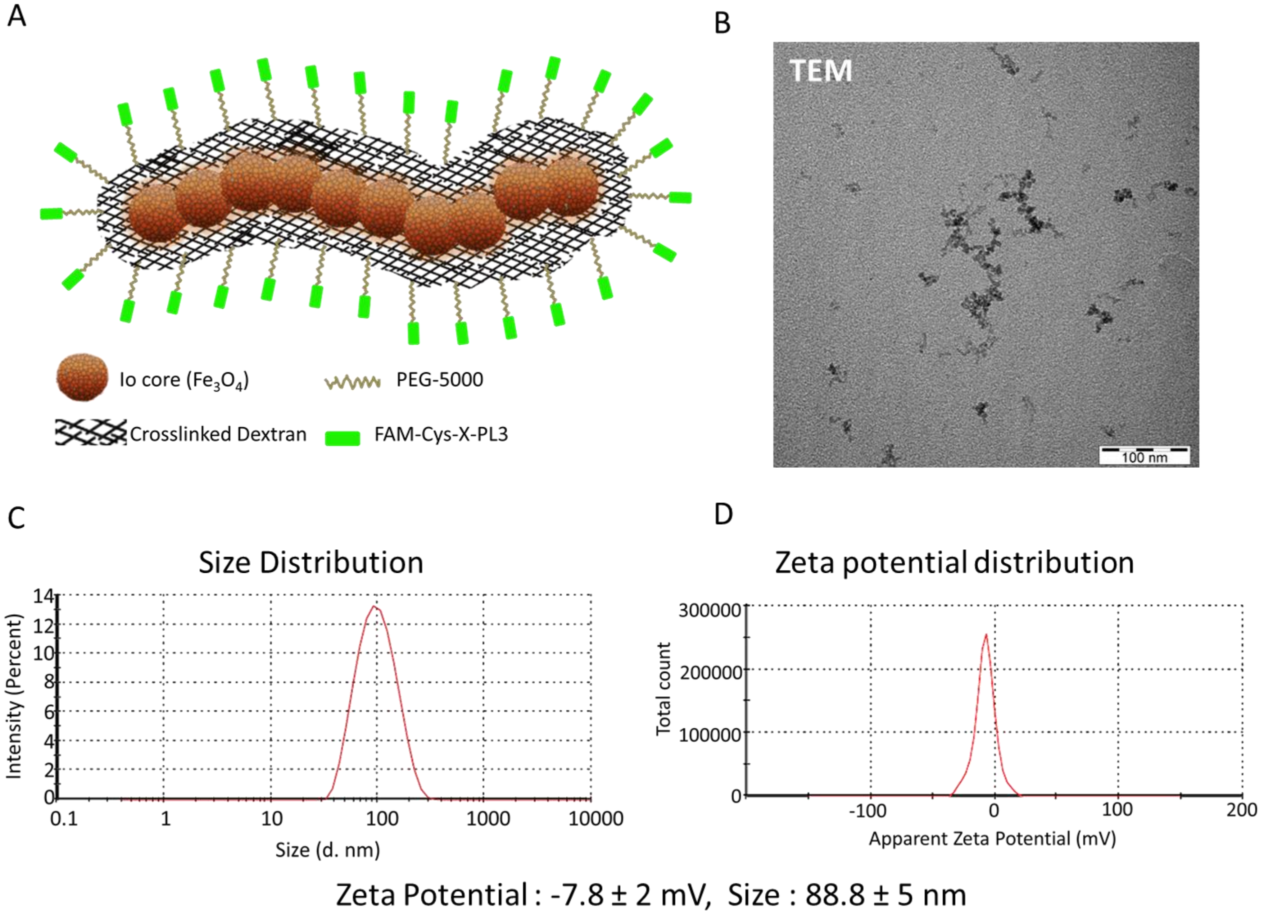

Figure. S6. Characterization of NWs. (A) Schematic representation of FAM-PL3-NWs. (B) Representative transmission electron micrograph of NWs. Scale bar:100 nm. (C, D) Size distribution of NWs and zeta potential as measured by DLS.

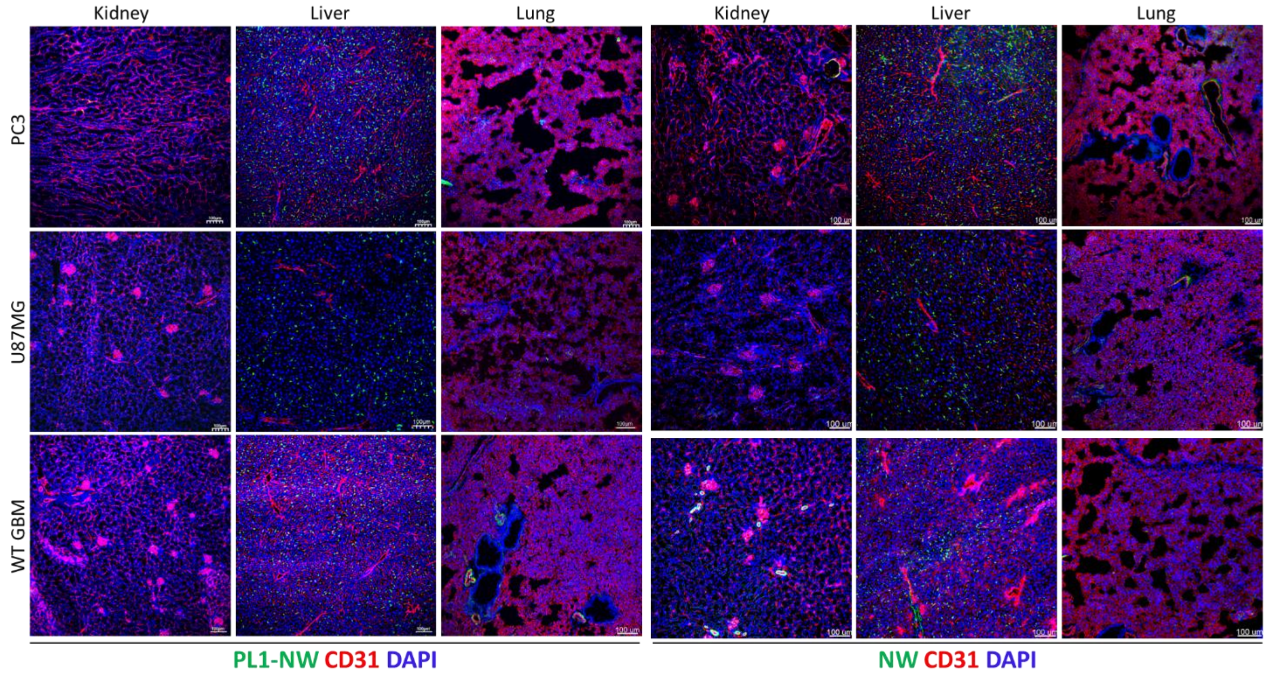

Figure. S7. The biodistribution of PL3-NWs in healthy organs. FAM-labeled PL3-NWs (or non-peptide NWs) were i.v. injected at 7.5 mg/kg into mice bearing PC3, U87, and WT GBM tumors. Five h after the injection, the mice were perfused through the heart with DMEM, and the tumors and control organs were collected. Sections of control organs were immunostained with antibodies to FAM (green), CD31 (red) and nuclei were counterstained with DAPI (blue). N=4; scale bar: 100 μm.

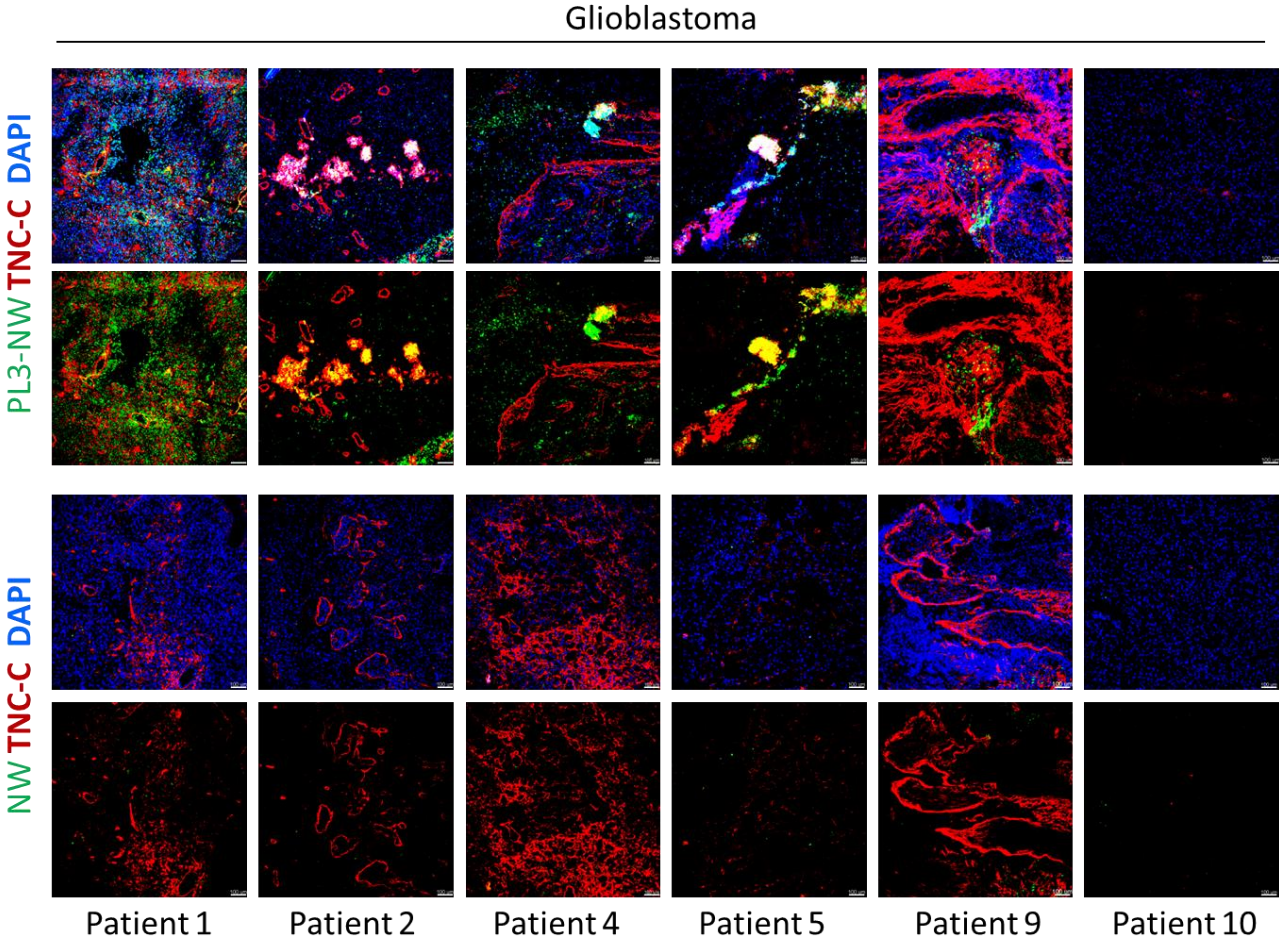

Figure. S8. PL3-NWs bind to surgical explants of human glioma tissues 1.

The snap-frozen clinical glioma tissues were sectioned and incubated with FAM-labeled PL3-NWs or non-targeted NWs, stained with anti-FAM and anti-TNC-C antibodies. Scale bar, 100 µm for all panels, tissues were stained for FAM (FITC, green), anti-TNC-C mouse (red), and nucleus (DAPI, blue).

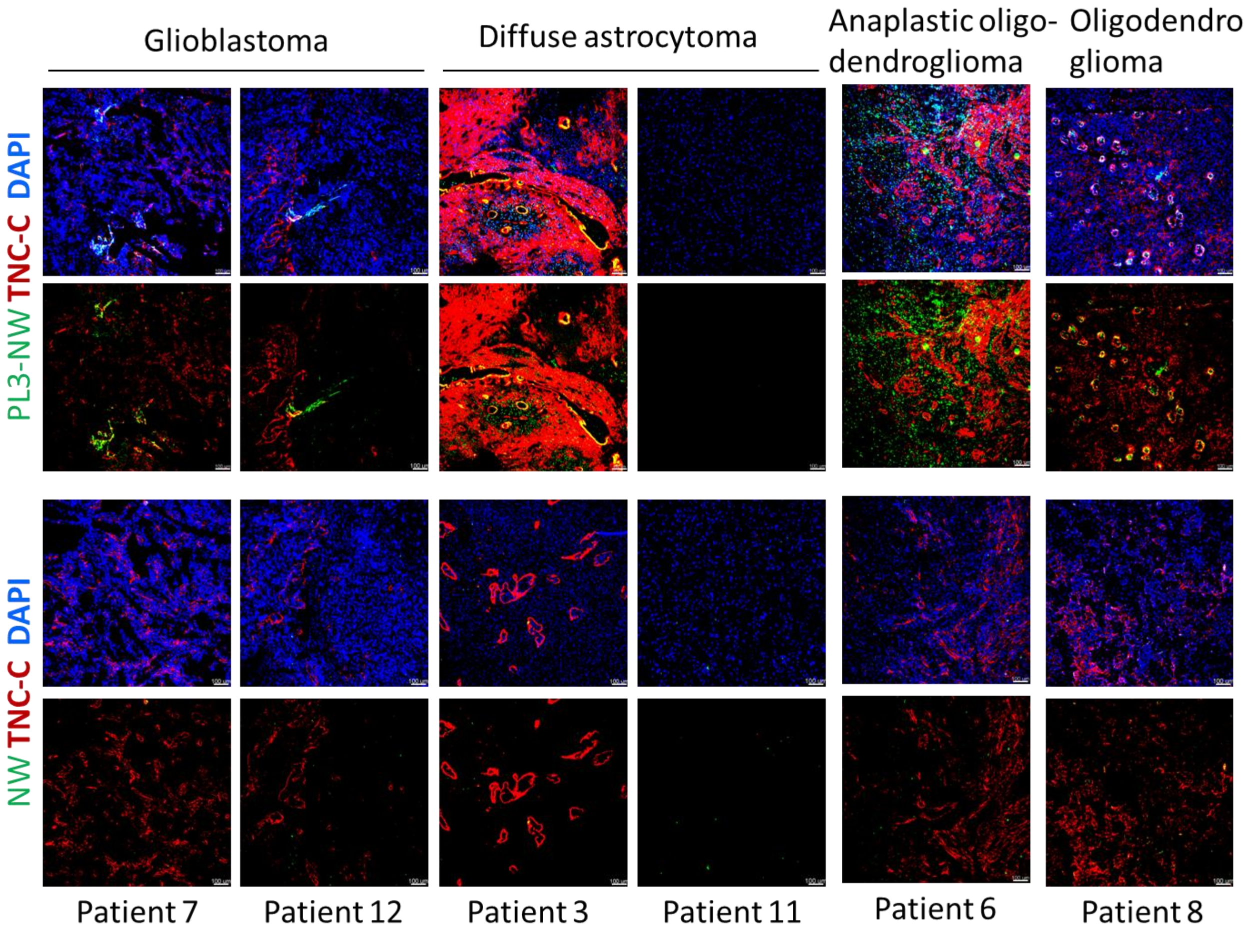

Figure. S9. PL3-NWs bind to surgical explants of human glioma tissues 2. The snap-frozen clinical glioma tissues were sectioned and incubated with FAM-labeled PL3-NWs or non-targeted NWs, stained with anti-FAM and anti-TNC-C antibodies. Scale bar, 100 µm for all panels, tissues were stained for FAM (FITC, green), anti-TNC-C mouse (red), and nucleus (DAPI, blue).

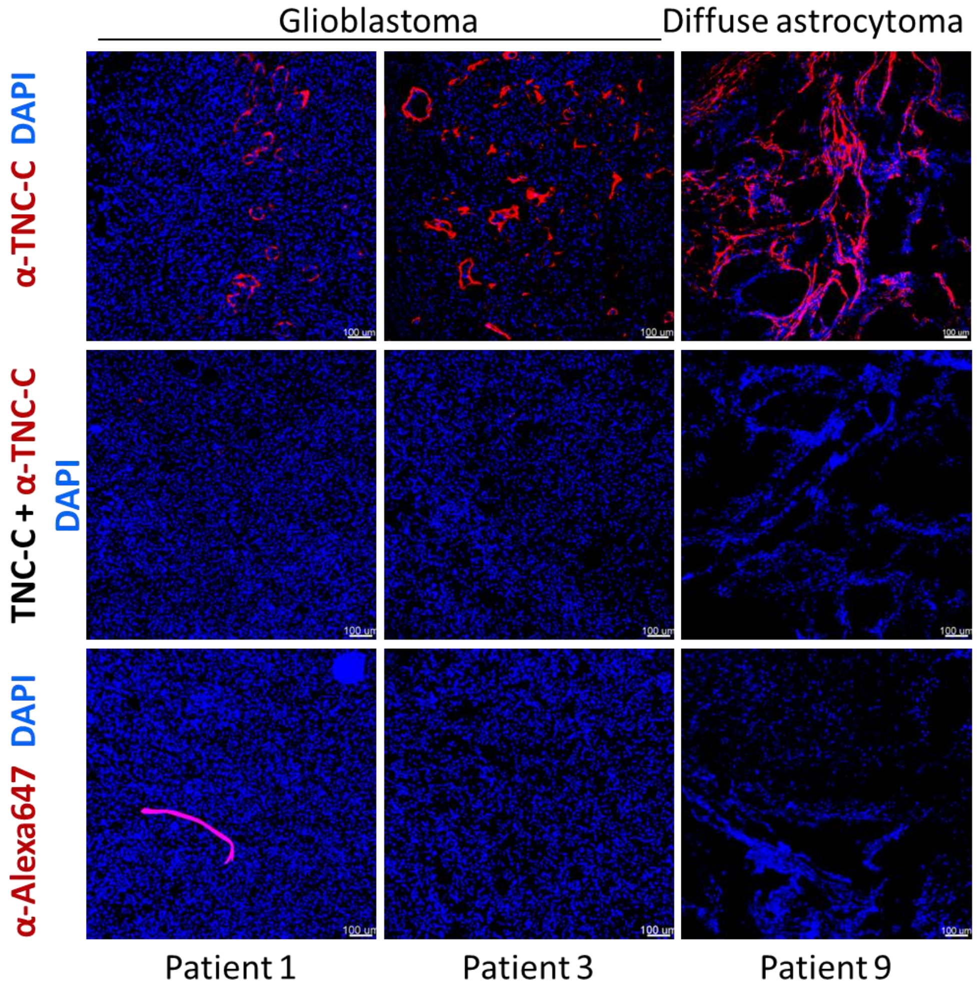

Figure. S10. Anti TNC-C antibody validation on surgical explants of human glioma tissues. The snap-frozen individual patient glioma tissues were sectioned and incubated with the anti-TNC-C mouse antibody (α-TNC-C), or α-TNC-C antibody (10 µg) is pre-incubated of TNC-C protein (60µg) before staining, or only secondary mouse α-alexa647 antibody. The tissue sections were examined by confocal microscopy. The top panel shows α-TNC-C antibody staining. The middle panel shows staining of α-TNC-C antibody is pre-blocked with recombinant TNC-C protein and reveals its specificity. The bottom panel shows secondary antibody staining control. Scale bar, 100 µm for all panels, tissues were stained for the anti-TNC-C mouse (red) and nucleus (DAPI, blue).

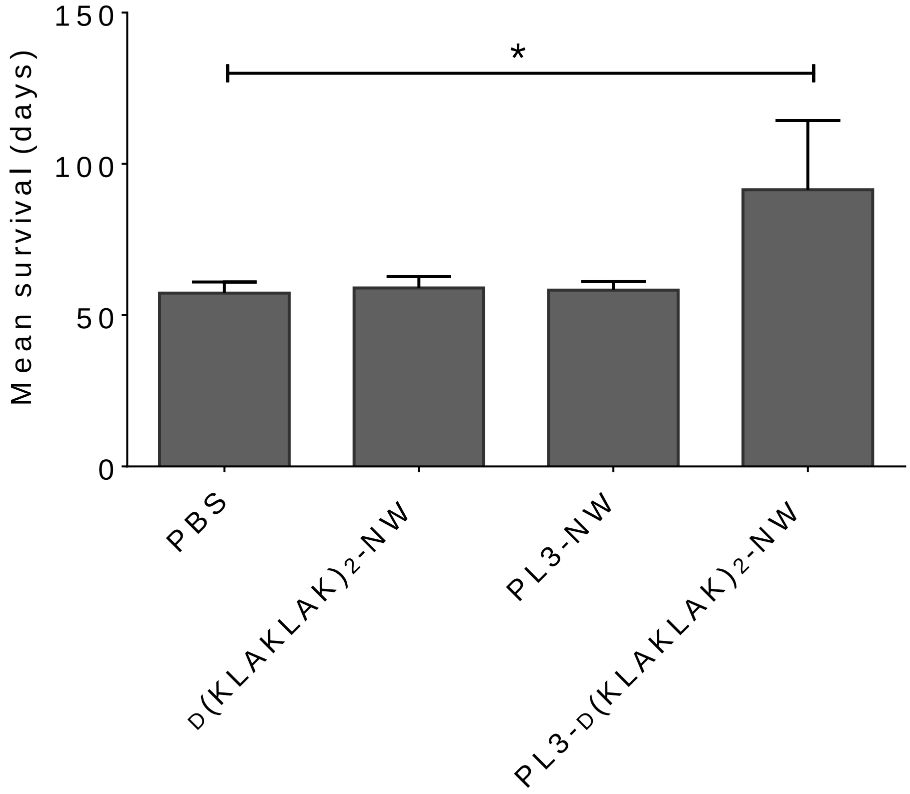

Figure. S11. Experimental tumor therapy with PL3-guided NWs extends survival of glioblastoma.

Mice bearing s.c. U87-MG mice were treated with 5 mg/kg of D(KLAKLAK)2-NWs, PL3-NWs, and PL3-D(KLAKLAK)2-NWs, or control PBS (N=6 mice/group). At the endpoint of the study (tumor volume >2000 mm^3), the mice were sacrificed by perfusion, and organs and tumors were collected. The mean survival of the different treatment groups was calculated. N = 6 mice/group; Error bars, mean ± SEM; Statistical analyses were performed using paired non-parametric test (Wilcoxon matched-pairs signed-rank test) or Friedman test in GraphPad Prism (*$p < 0.05$).

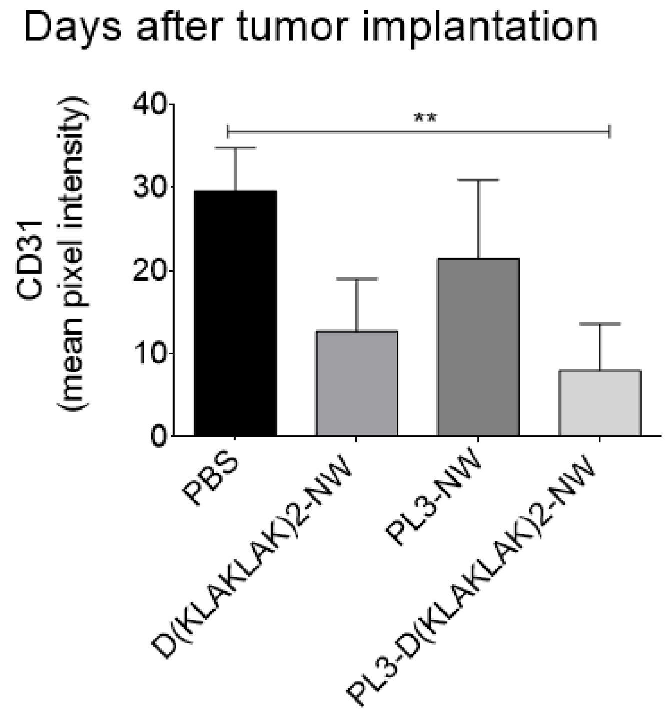

Fig. S12. Analysis of CD31 immunofluorescence signal in post-treatment tumors by quantitative analysis of FAM fluorescence in a series of images by Fiji ImageJ. Tumors from animals treated with PL3-$_{D}$(KLAKLAK)$_2$-NWs showed a statistically significant reduction in vascularization compared to other groups. Student's unpaired t-test; the error bars: mean ± SEM; ** $p < 0.01$. N=3.

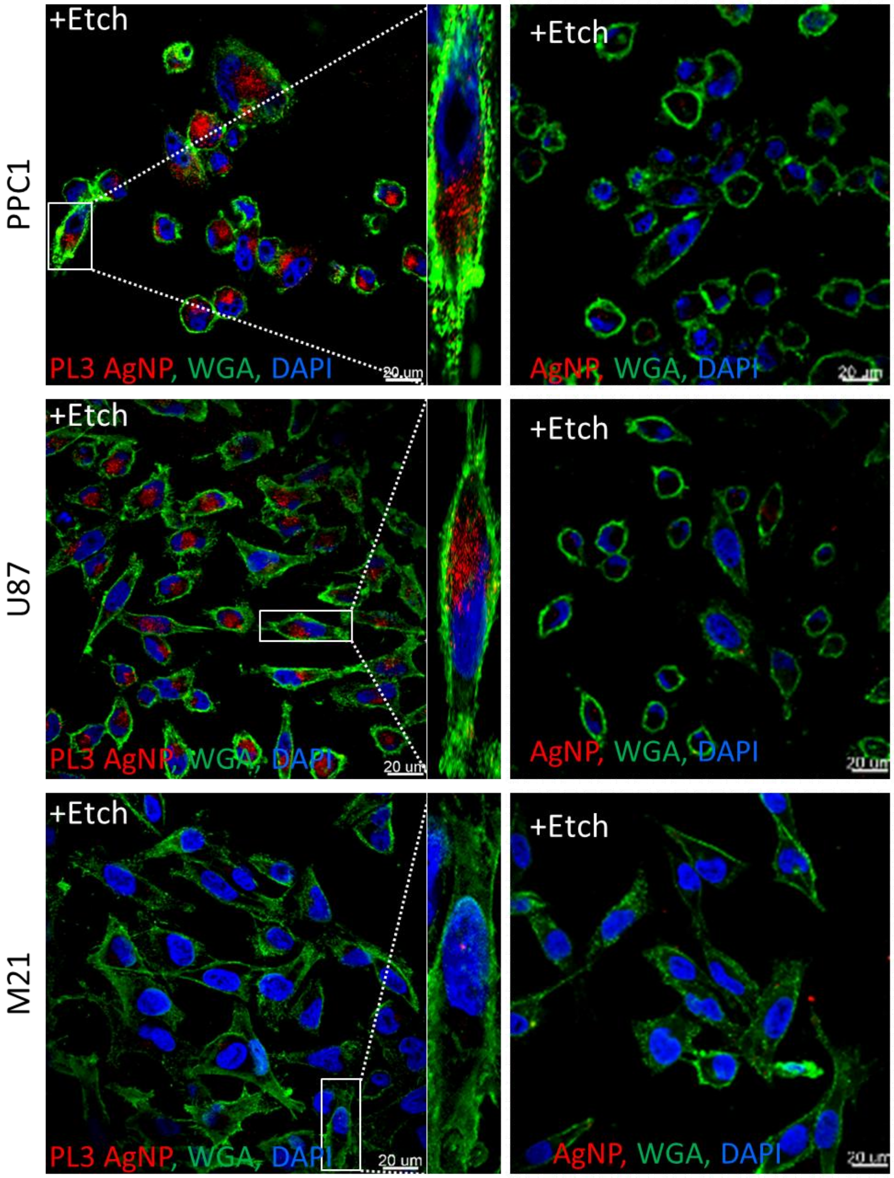

Fig. S13. NRP-1-dependent internalization of PL3-functionalized AgNPs. PL3-AgNPs, or control AgNP particles (37 µL of 100 O.D. stock solution) labeled with CF555 fluorophore were incubated with PPC1 prostate carcinoma, U87MG glioma, or M21 melanoma cells for 1 hour, washed, treated with an optional etching solution to dissolve extracellular particles, and processed for confocal imaging. (A) Confocal imaging of cells. Note robust uptake of PL3-AgNPs (red) to NRP-1-positive PPC1 and U87MG (and not NRP-1 negative M21) cells (magnified cell image shown in the box on the right of each image). In contrast, control particles did not bind to the cells independent of their NRP-1 expression status (insets). Scale bar: 20µm (main images), and 2µm (insets). Etching had only a modest effect on PL3-AgNP signal in PPC1 and U87MG cells, suggesting that most of the particles were internalized and protected from etchant by the cellular membrane.

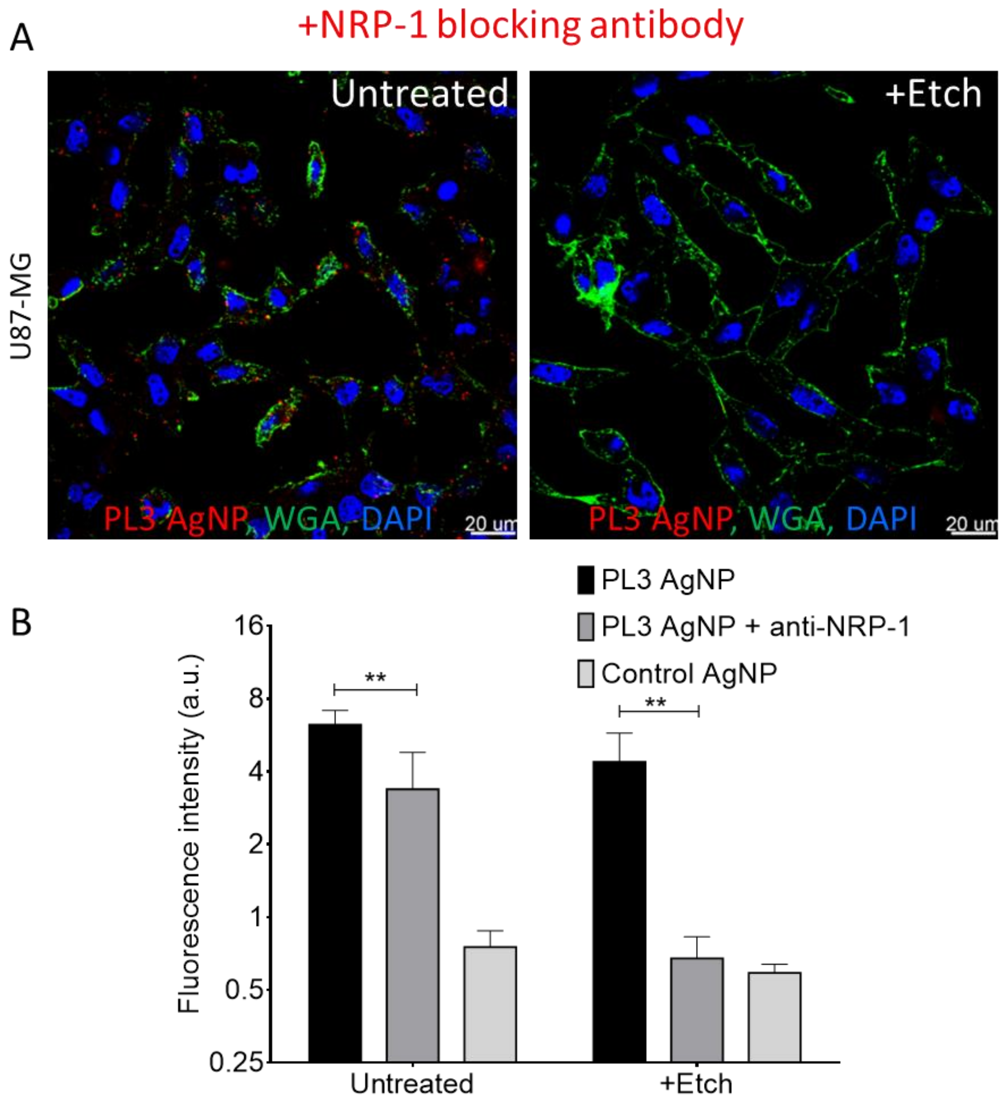

Fig. S14. NRP-1-dependent internalization of PL3-functionalized AgNPs in U87-MG cells (A) U87-MG cells were grown on coverslips and preincubated for 1 hour with NRP1 blocking antibody (30 µg/ml), followed by incubation with PL3 AgNPs (red). Plasma membrane was stained with wheat germ agglutinin (green) and the nuclei with DAPI (blue). Optionally, extracellular AgNPs were dissolved by etching. Representative immunofluorescence images are shown. Scale bars, 20 µm. (B) Quantitation of binding and internalization of CF555-labeled AgNPs using ImageJ for samples from (A) and at least 3 independent experiments were carried out. Error bars: mean ± SD (N=6), P-values were determined using unpaired Student's t-test (** $P \leq 0.01$).

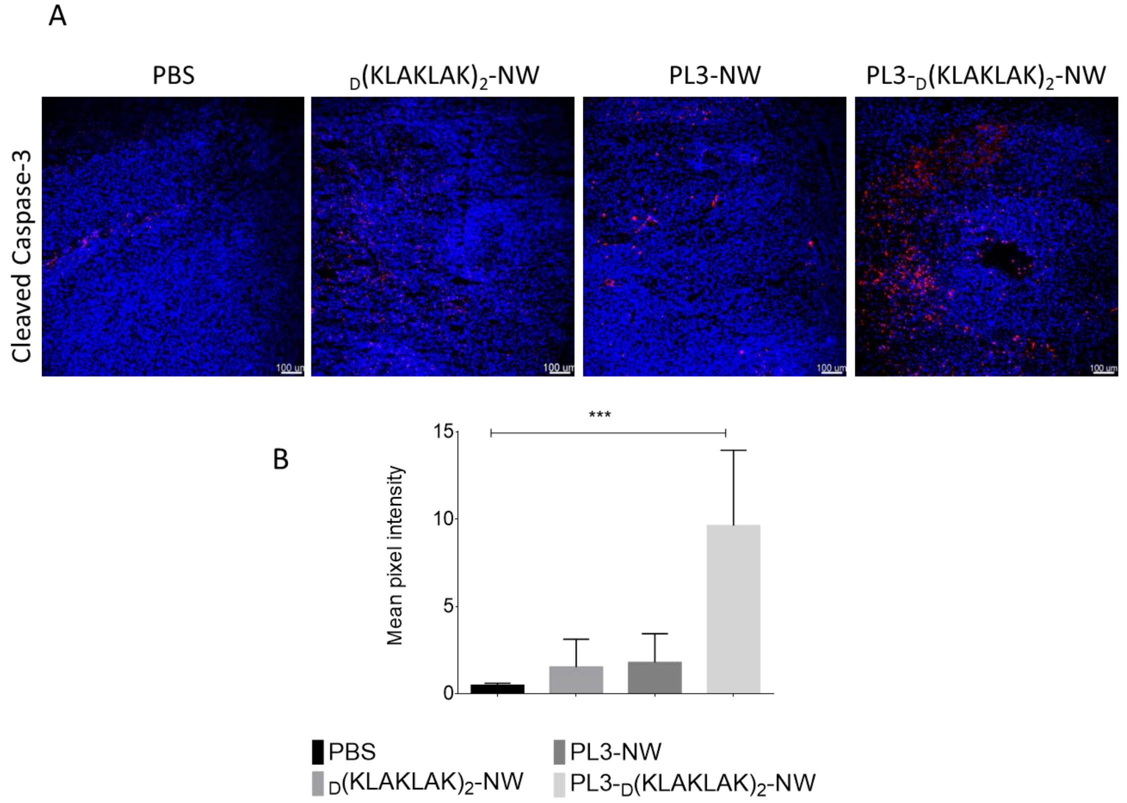

Fig. S15. Analysis of cleaved Caspase-3 immunofluorescence signal in post-treatment tumors. Tumors from animals treated with PL3-D(KLAKLAK)2-NWs showed a statistically significant increase in apoptotic cells compared to other groups. Student's unpaired t-test; the error bars: mean ± SEM; *** $P \leq 0.001$. N=3.

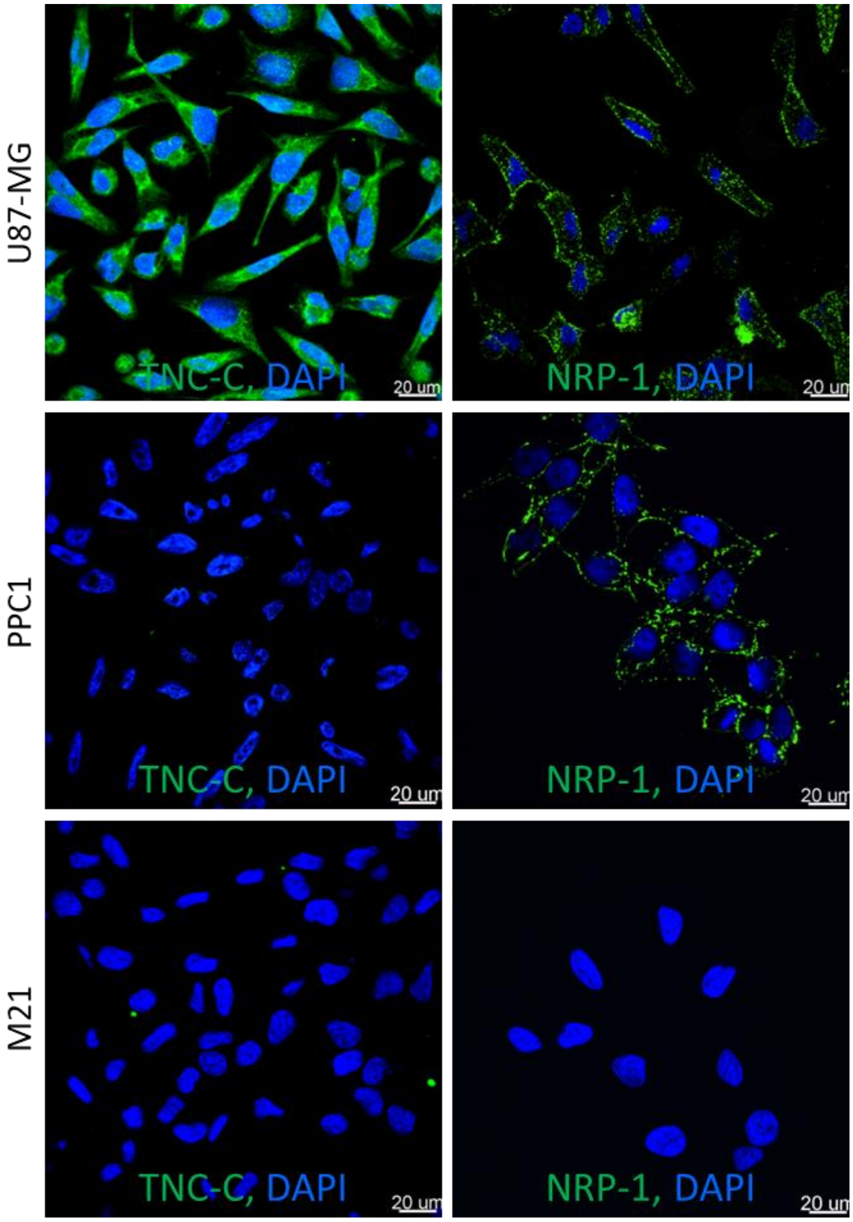

Fig. S16. TNC-C and NRP1 receptor expression in U87-MG, PPC1, and M21 cells. The cells grown in coverslip, the cells were stained for TNC-C (green) and NRP1 (green) receptors with FAM-ScFV G11 TNC-C antibody and rabbit α-NRP1 antibody. The nuclei were stained with DAPI(blue). Representative immunofluorescence images from three independent experiments are shown. Scale bars, 20 µm.

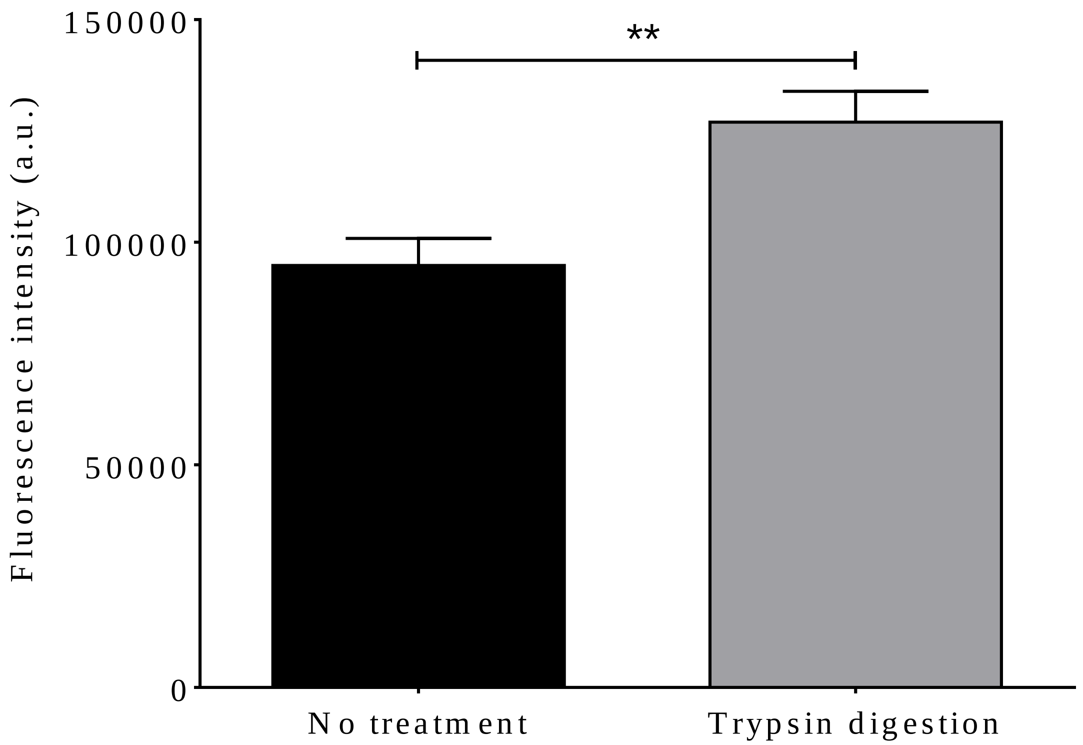

Fig. S17. Dequenching of FAM-PL3 upon tryptic digestion of FAM-PL3/TNC-C complex. The Ni-NTA Magnetic Agarose Beads (QIAGEN, Hilden, Germany) were coated with His-6X tagged TNC-C (300 µg/100 µl beads) at room temperature for 1 h in 400µl of PBS. The TNC-C beads were washed 3 times with washing buffer (PBS, 0.05% NP-40, 1% BSA), followed by incubation with FAM-PL3 peptide (200 µg in 400µl in washing buffer) at room temperature for 2 h. The unbound peptides were removed by rinsing 6 times with washing buffer, and the bound FAM-PL3/TNC-C complex was eluted with 1ml of PBS containing 500mM Imidazole and 0.1% NP40. To measure the dequenching effect, the FAM-PL3/TNC-C complex was treated with trypsin (2.5mg/mL) for 4 h at 37°C. The intensity of fluorescence of the untreated and trypsin-treated complex was measured by VICTOR Multilabel Plate Reader (PerkinElmer) at 488/516nm. Student's unpaired two-tailed t-test; the error bars: mean ± SD; ** $p < 0.01$, N=3.
